# Persistent Halogenated Organic Pollutants in Deep-Water-Deposited Particulates from South China Sea

**DOI:** 10.3390/toxics11120968

**Published:** 2023-11-29

**Authors:** Jia-De Lee, Tsyr-Huei Chiou, Hong-Jie Zhang, How-Ran Chao, Kuang-Yu Chen, Yan-You Gou, Chien-Er Huang, Sheng-Lun Lin, Lin-Chi Wang

**Affiliations:** 1 Department of Environmental Science and Engineering, National Pingtung University of Science and Technology, Neipu, Pingtung 91201, Taiwan; jdlee01251212@gmail.com (J.-D.L.); power20342000@gmail.com (Y.-Y.G.); 2 Department of Life Sciences, National Cheng Kung University, Tainan 701, Taiwan; thchiou@mail.ncku.edu.tw; 3 School of Mechanical Engineering, Beijing Institute of Technology, Beijing 100811, China; 3220210320@bit.edu.cn; 4 Center for Agricultural, Forestry, Fishery, Livestock and Aquaculture Carbon Emission Inventory and Emerging Compounds, General Research Service Center, National Pingtung University of Science and Technology, Neipu, Pingtung 91201, Taiwan; 5 Institute of Food Safety Management, College of Agriculture, National Pingtung University of Science and Technology, Pingtung 91201, Taiwan; 6 School of Dentistry, College of Dental Medicine, Kaohsiung Medical University, Kaohsiung 807, Taiwan; 7 Department of Occupational Safety and Health, Faculty of Public Health, Universitas Airlangga, Kampus C, Mulyorejo, Surabaya 60115, Indonesia; 8 National Applied Research Laboratories, Taiwan Ocean Research Institute, Kaohsiung 852, Taiwan; littlefish3@short.idv.tw; 9 Department of Mechanical Engineering, Institute of Mechanical Engineering, Cheng Shiu University, Niaosong District, Kaohsiung 833, Taiwan; k1668@gcloud.csu.edu.tw; 10 Super Micro Mass Research & Technology Center, Cheng Shiu University, Niaosong District, Kaohsiung 833, Taiwan; 11 Department of Environmental Engineering, National Cheng Kung University, Tainan 701, Taiwan; z1120811@ncku.edu.tw; 12 Department of Marine Environmental Engineering, National Kaohsiung University of Science and Technology, Nanzih District, Kaohsiung 81157, Taiwan

**Keywords:** deposited particulate, marine pollution, persistent organic pollutants (POPs), polybrominated diphenyl ethers (PBDEs), mass flux

## Abstract

POP data are limited in the marine environment; thus, this study aimed to investigate background persistent organic pollutant (POP) levels in oceanic deep-water-deposited particulates in the South China Sea (SCS). Six POPs, including polychlorinated dibenzo-*p*-dioxins/dibenzofurans (PCDD/Fs), dioxin-like polychlorinated biphenyls (DL-PCBs), polybrominated diphenyl ethers (PBDEs), polybrominated dibenzo-*p*-dioxins/dibenzofurans (PBDD/Fs), polychlorinated diphenyl ethers (PCDEs), and polybrominated biphenyls (PBBs), were investigated in eight pooled samples from the SCS from 20 September 2013 to 23 March 2014 and 15 April 2014 to 24 October 2014 at depths of 2000 m and 3500 m. PBDEs were the most predominant compounds, with the highest mean Σ_14_PBDE of 125 ± 114 ng/g dry weight (d.w.), followed by Σ_17_PCDD/F, Σ_12_PBDD/F, and Σ_12_DL-PCB (275 ± 1930, 253 ± 216, and 116 ± 166 pg/g d.w., respectively). Most PBDD/F, PBB, and PCDE congeners were below the detection limits. PCDDs had the highest toxic equivalency (TEQ), followed by PBDDs and DL-PCBs. Among the six POPs, PBDEs were the major components of the marine-deposited particles, regarding both concentrations and mass fluxes. Compared to 3500 m, PBDE levels were higher at a depth of 2000 m. PBDE mass fluxes were 20.9 and 14.2 ng/m^2^/day or 68.2 and 75.9 ng/m^2^/year at deep-water 2000 and 3500 m, respectively. This study first investigated POP levels in oceanic deep-water-deposited particles from existing global data.

## 1. Introduction

Polychlorinated dibenzo-*p*-dioxins and dibenzofurans (PCDD/Fs), dioxin-like polychlorinated biphenyls (DL-PCBs), polybrominated diphenyl ethers (PBDEs), polybrominated dibenzo-*p*-dioxins and dibenzofurans (PBDD/Fs), polychlorinated diphenyl ethers (PCDEs), and polybrominated biphenyls (PBBs) are considered persistent organic pollutants (POPs), which are a bioaccumulative class of compounds that can withstand biological, chemical, and physical degradation [1]. These six POPs have been observed in different environmental media such as atmospheric air, airborne particulate matter (PM), aquatic PM and sediments, soil, surface water, and groundwater [2,3,4,5,6,7,8]. Our previous study investigated PCDD/Fs, PCBs, PBDEs, PBDD/Fs, PBBs, and PBDEs in the atmosphere of the Pacific Ocean near Taiwan and the Philippines, where we observed extremely high PBDE levels compared to other POPs [2]. The ocean is recognized as the final sink for POPs [2,9], and accumulation of organic matter, PM, and fine sediments in the aquatic environment is associated with high organic carbon partition coefficients (*K_oc_*) [10]. POPs can be transported by any particle and organic matter movements, and these movements can transport POPs to even greater ocean depths [9,11]. The physiochemical properties of POPs contribute to their geo-accumulation in ocean particles and sediments [12], and the lipophilicity of POPs also assists their accumulation in the marine biota [13]. These POPs also easily and persistently enter several biological media through bioaccumulation and biomagnification to accumulate in human tissue, such as blood [14,15], serum [16,17], breast milk [18,19], and lipids [15,20], further inducing hormone disruptions, carcinogenicity, and reproduction and neurodevelopment toxicity [2,21].

The dioxin-like compounds known as “PCDD/Fs” commonly originate from anthropological activities [22] such as municipal and industrial combustion discharges, specifically from manufacturing plants that utilize chlorinated compounds (e.g., trichlorophenol) such as paper pulp bleaching, metallurgical process, and herbicide and pesticide production plants [23,24,25,26,27,28]. The dioxin-like toxicity of DL-PCBs is attributed to their ability to rotate and adopt a coplanar structure that is similar to that of dioxins [26]. DL-PCBs are manufactured as mixtures and widely used as ingredients in flame retardants, coatings, inks, and paints [29,30]. PBDEs are structurally similar to PCBs and are only used as brominated flame retardants (BFRs) in the manufacture of electronic appliances and devices, textiles, coatings, and household furniture [31]. PBDD/Fs are structurally similar to PBDEs, formed and released through the pyrolysis of commercial BFR mixtures [32,33,34], and have been observed on the surfaces of BFR-containing products [2,35,36]. In our previous studies, PBDD/Fs were also found in indoor PM_2.5_ [37] and indoor dust [33,37]. PCDEs are similar to PCBs in structure and are used as flame retardants, hydraulic fluids, electric insulators, plasticizers, and lubricants [38]. They are by-products of municipal waste incinerator flues, technical chlorophenols, chlorinated phenoxyacetic acids, and incomplete combustion media [39,40]. PCDEs mainly exist as impurities in chlorophenol preparations [41,42]. Unlike PCBs, which are versatile, PBBs are only used as flame retardants in polymeric resins and only have one commercially available form: FireMaster^®^ [43].

Several studies have reported the presence and environmental fate of organohalogen POPs. Due to their non-polarity and high *K_oc_*, these substances have a similar dispersion and deposition on land. For example, they tend to be dispersed and deposited in sinks such as soils, watersheds, and oceans, where PCDD/Fs or DL-PCBs are then released into the atmosphere [4,28]. Additionally, PBDEs are quickly diffused from the surface of BFR-containing products and can bind to particles such as PM_2.5_ [32] and dust [33]. Few studies have addressed POPs in deposited particles in deep oceans. According to Long-Range Atmospheric Transport (LRAT), POPs have been detected in remote locations and places [44] that are far from anthropogenic activities, such as Antarctica [45,46,47], the Arctic [11,48], Tibetan plateaus [49], the oceanic atmosphere [2,50], and surface and deep ocean waters [51,52]. POPs can partition between the gas and particle phases due to their semi-volatile properties [53] and are deposited in dissolved form through dry deposition and particulate form through wet deposition [54]. Evidence of POP contamination in the marine environment has been reported in several works. POPs are further transported into deep ocean waters through the global thermohaline circulation [51]. POPs can accumulate in oceanic particles due to their physicochemical properties [9]. The persistent nature and bioaccumulative characteristics of POPs may result in their biomagnification through the food web [55,56]. Ultimately, most oceanic particles will be deposited into sediment, which can thus reveal historical pollution trends. Kobusińska et al. (2022) [57] made a stunning discovery that 50% of marine sediment samples’ PCDD/Fs levels exceeded the environmental limit in polar zones (Arctic and Antarctic), evidencing that the atmosphere mainly carries PCDD/Fs from central latitudes to the polar zones. Assefa et al. (2014) [58] modeled PCDD/F pollution trends in the sediment cores of Baltic Sea coverings over the last century, estimating the overall peak to be in 1994 (±5 years) and a sediment half-life of 29 ± 11 years, which was consistent with trends in European lakes as well as the declining levels of atmospheric PCDD/F levels. Ma et al. (2015) [59] have reported that PCBs in surficial sediments have higher concentrations in shallow water (<500 m in depth) than in deep water (>500 m in depth) of the Arctic Ocean. However, a high PCB concentration (931 to 4195 pg/g dw of Σ_36_PCBs) was observed in the southern Mariana Trench at water depths of 7000–11,000 m, far higher than that recorded in worldwide marine surface water sediments from shallower depths in previously published studies [60]. PCBs and PBDEs have also been reportedly identified in deep-sea sediments from the Indian ocean [22,61], while PBDEs have been detected in marine sediments from North America, Europe, Oceania, the Polar regions, the Middle East, and Asia [62]. In contrast to PCDD/F, PCB, and PBDE contaminants, studies of PBBs, PCDEs, and PBDD/Fs in marine or polar region sediments have been lacking over the last decade. In a Norwegian study [60], BB-153 was the only investigated PBB congener, and its levels were below the detection limit (<0.00023 ng/g dry weight (d.w.)) in all sediment samples from the Barents and Pechora Seas [63]. Regarding PCDEs, Koistinen et al. (1997) [64] investigated their levels in sediments collected from the Gulf of Finland, and they were found to be almost negligible. In another study investigating marine surface sediments in the coastal waters of Hong Kong and Korea, PBDD/F concentrations were twice as low as their chlorinated counterparts [65].

Our study areas are located in the South China Sea (SCS) adjacent to the west Pacific Ocean. The SCS is bordered by several countries which are potential sources of POPs and are considered as centers of e-waste recycling and ship dismantling industries [66,67,68,69,70]. The SCS may be contaminated with POPs through surface runoff, atmospheric deposition, and rain washes, which leads to POP biomagnification in the marine biota [67]. Currently, there is still a lack of studies concerning POP concentrations in deposited particulate samples collected from the SCS. In this study, we measured the concentrations, TEQ levels, and mass fluxes of six POPs in our 1-year investigation of marine-deposited particle samples from the SCS. Then, we attempted to discuss the influence of controlling factors and the LRAT process on the POP distribution, which may also provide a better understanding of the environmental fate of these POPs and their global impact.

## 2. Materials and Methods

### 2.1. The Collection of Vertically Deposited Particulate in Seawater

**Figure 1:**
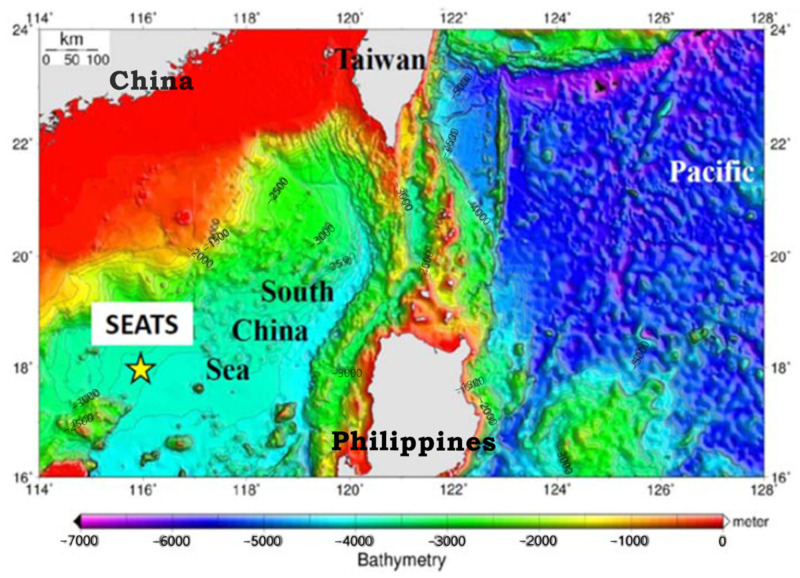
The location of the SEATS site (18° N, 116° E) was near a two-series anchorage-type sediment trap in the South China Sea. The locations of SEAT 1 and SEAT 2 are 17°59.685′ N, 116°00.1408′ E and 18°06.3852′ N, 116°01.9695′ E, respectively. This is a 1-year survey between 20 September 2013–23 March 2014 and 15 April 2014–24 October 2014.

This is a 1-year environmental survey for determining POPs on the marine deposited particulate in SCS. The sampling location was near the South East Asia Time Series (SEATS) monitoring site (18° N, 116° E) in the SCS, as shown in Figure 1. A two-series anchorage-type sediment trap set (Technicap Sediment Trap PPS 5/2, La Turbie, France) was installed to collect the vertical particulate deposits in deep water at 2000 m (m) and 3500 m, respectively (with a seabed depth of about 3800 m), between 20 September 2013 and 23 March 2014 at SEATS 1 (17°59.685′ N, 116°00.1408′ E) and between 15 April and 24 October 2014 at SEATS 2 (18°06.3852′ N, 116°01.9695′ E) (Figure S1 in the Supplementary Materials). For the size of the trap body, it has a diameter of centimeters (cm), a height of 230 cm, a collection area of 1.00 m^2^, and a weight of 110 kg (kg) in the ambient air and 45 kg in the water, respectively. The trap body was connected to a rotation container and 24 bottles (250 mL), with a 12-Volt AA alkaline battery pack for a battery life of at least 18 months. The rotation container could be controlled via a computer to gather a sample bottle every 8 days. The solvent in the selected bottle was a mixture of 200 g (g)/L NaCl and 1 g/L HgCl_2_, which was used to collect vertically deposited particulates at 2000 or 3500 m depths. The sediment trap set was recovered after 6 months, and 48 samples were collected (24 samples from the 2000 m trap and 24 examples from the 3500 m trap) and stored at 4 °C.

### 2.2. Extraction, Cleanup, and Chemical Analysis

After the freeze-drying process, all the deposited particulates were prepared for pretreatment before chemical analyses. The extraction, cleanup, and the analytical method procedures for the samples followed those used in previous studies to analyze halogenated POPs in the environment [2,71] (Figure S2). Briefly, an identified amount of surrogates and standards was pre-labeled with isotopes (please see Tables S2–S10 in the Supplementary Materials) and spiked to verify the sampling process’s collection efficiency before analysis. After collecting the deposited particulate samples, samples were spiked with internal standards to ensure that they were well mixed with toluene before Soxhlet extraction. These samples were used to monitor the extraction and cleanup procedures. The sample mixture was extracted in a Soxhlet extractor with toluene for 24 h. The extracts were sequentially concentrated and treated with concentrated sulfuric acid. They underwent a series of sample cleanup and fractionation procedures via a multi-layered silica column, an alumina column, and an activated carbon column. Biphenyl compounds, such as non-planar PCBs and PBBs, were eluted with 15 mL hexane during the alumina column cleanup. An activated carbon column was used for further cleanup procedures. After the elution of 25 mL dichloromethane/hexane (1/24, *v*/*v*), the activated carbon column was sequentially eluted with 5 mL toluene/methanol/ethyl acetate/hexane (1/1/2/16, *v*/*v*) for PCDEs, PBDEs, planar PCBs, and PBBs, which was followed by 40 mL of toluene for PCDD/Fs and PBDD/Fs. Planar and non-planar PCB/PBB eluates were mixed to represent the PCB and PBB samples. The eluate was concentrated to approximately 1 mL and transferred to a vial. The concentrated eluate was further concentrated to near dryness using a stream of nitrogen. Immediately before injection, 10 μL of standard solution for recovery checking was added to the sample extract to minimize the possibility of sample loss.

### 2.3. Instrumental Analysis

Sixty-six halogenated POPs, including seventeen 2,3,7,8-substituted PCDD/Fs, twelve 2,3,7,8-substituted PBDD/Fs, twelve DL-PCBs, five PBBs, fourteen PBDEs, and six PCDE congeners, were analyzed via high-resolution gas chromatography–high-resolution mass spectrometry (HRGC/HRMS). Detailed instrumental analysis parameters for the PCDD/Fs, PCBs, PBDEs, PBDD/Fs, PBBs, and PBDEs were given in our previous study [71]. The HRGC equipment (Hewlett–Packard 6970 Series gas, Palo Alto, CA, United States) was equipped with a silica capillary column (J&W Scientific, Santa Clara, CA, United States) (a detailed description of GC columns and temperature conditions can be found in Table S1 in the Supplementary Materials) and a splitless injector, whereas the HRMS equipment (Micromass Autospec Ultima, Manchester, UK) was fitted with a positive electron impact (EI+) source. Selected ion monitoring (SIM) was used with a resolving power of 10,000. The electron energy and source temperature were specified as 35 eV and 250 °C, respectively.

For calculating the toxic equivalency (TEQ) values of PCDD/Fs, the World Health Organization (WHO) toxic equivalent factor (TEF) schemes of PCDD/Fs were used for chlorinated analogues (2005 World Health Organization toxic equivalent factors (WHO_2005_-TEF)) [72]. Considering the relative effect potency (REP) data in in vivo mammalian models, Dr. Van den Berg and his team of workers recommended the same WHO-TEF values for PCDD/Fs and PBDD/Fs to assess their impacts on humans based on similar mammalian REP values for PBDD/Fs and PCDD/Fs [73]. Sampling and analyses quality assurance and quality control (QA/QC) followed the U.S. EPA method 1614 and Taiwanese EPA NIEA M803.00B. Table S1 lists the GC column parameters and the temperature conditions of the GC oven. The recoveries of precision and recovery (PAR), the surrogate, internal, and alternate standards; and the spiked concentrations of the surrogate and internal standards for the 6 POPs met the acceptable criteria in Tables S2–S7. The blanks (BKs), including the Filter BK, Store BK, and Sea BK, were regularly examined in each batch of the analytic samples (please see Tables S8 and S9 in the Supplementary Materials). The limits of detection (LOD) values (signal-to-noise (S/N) > 3) and limits of quantification (LOQs) (S/N > 10) were also determined in this study. The method detection limits (MDLs) were calculated as 2.5–5.0 times the estimated LODs. The MDLs of PBDEs, PBBs, and PCDEs ranged from 0.0730 to 46.0 pg/g d.w., except for BDE-209, for which it was 330 pg/g d.w. The MDLs of PCDD/Fs, DL-PCBs, and PBDD/Fs were 0.0470–25.7 pg/g d.w. (Table S10).

### 2.4. Statistical Analysis

To avoid underestimation of PBBs, PCDEs, and certain PBDD/Fs, POP measurements that were below the MDLs were set as 1/2 the MDL. Descriptive analysis was used to determine the means and standard deviations (SDs) of PCDD/Fs, PBDD/Fs, DL-PCBs, and PBDEs at water depths of 2000 or 3500 m. All measurements of PCDD/Fs, PBDD/Fs, DL-PCBs, and PBDEs at the eight locations were not normally distributed, and the sample size was small. POP levels at two different deep-water depths, i.e., 2000 and 3500 m, were examined by Mann–Whitney *U* and Wilcoxon nonparametric tests. Analyses were carried out using the Statistical Package for Social Sciences (SPSS) version 12.0.

## 3. Results

**Figure 2:**
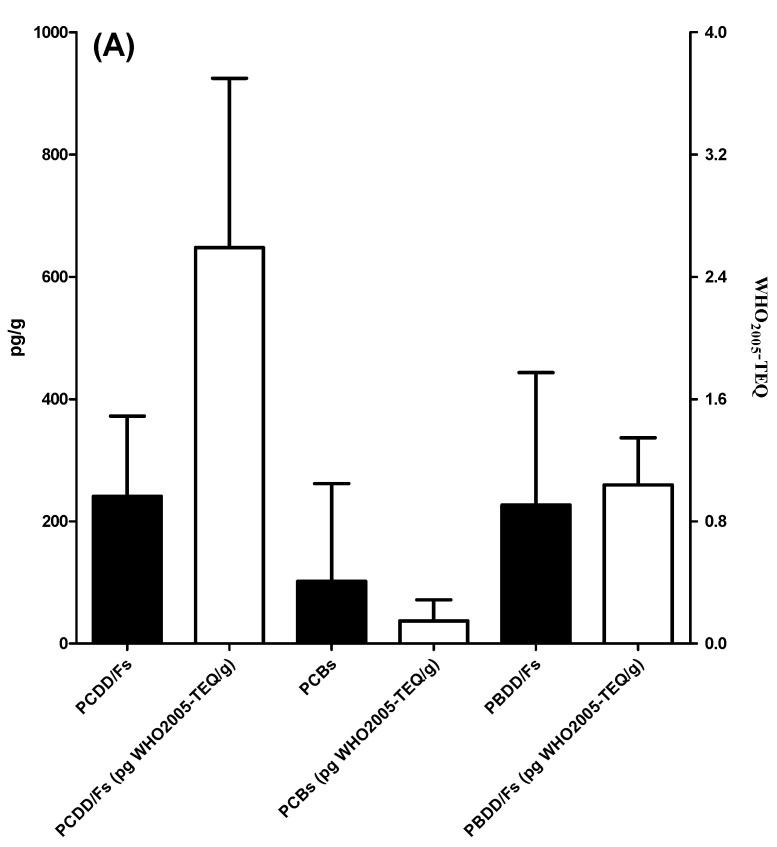

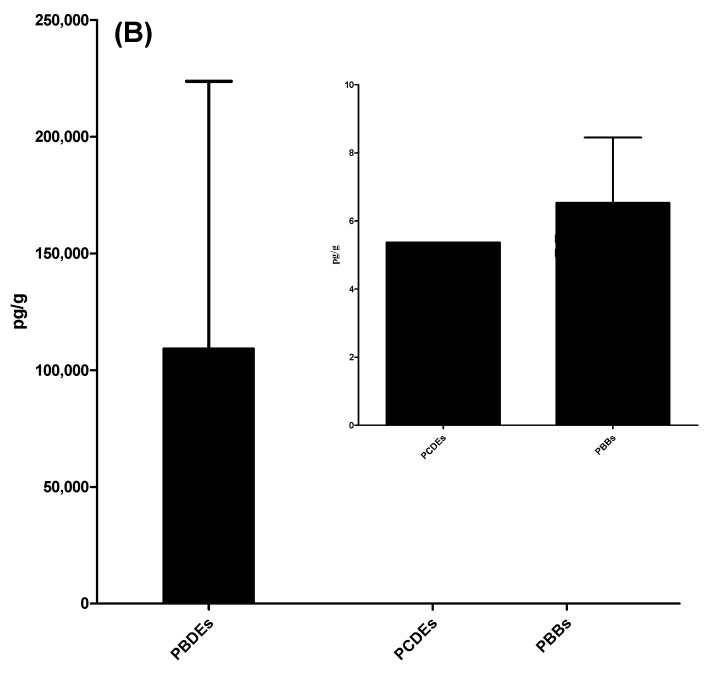
The concentrations of the six POPs in deposited particulate samples: (**A**) PCDD/Fs, PCBs, and PBDD/Fs; (**B**) PBDEs, PCDEs, and PBBs.

**Figure 3:**
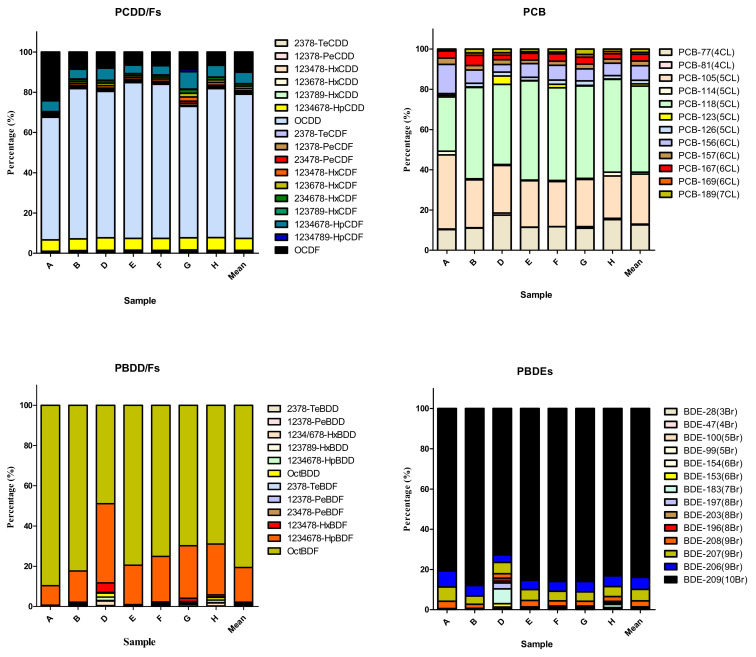
Characteristic patterns of the halogenated POPs were shown in the marine deposited particles. The letters of A, B, D, E, F, G, and H means different sampling periods and locations (SEAT 1 and SEAT 2) in accordance with the Table 1. The composition of sample C was excluded due to all measurements of POPs below MDLs.

Oceanic particulate samples deposited over 1 year and collected by the sediment trap at the two SEATS sites were analyzed for the presence of six POPs: PCDD/Fs, PCBs, PCDEs, PBDD/Fs, PBBs, and PBDEs (see Table 1 and Supplementary Materials Tables S11–S16). In general, the POP residue concentrations in deposited particulates followed the following order: PBDEs > PCDD/Fs ≥ PBDD/Fs ≥ PCBs ≥ PBBs ≥ PCDEs (Figure 2). PBDEs had the highest mean Σ_14_PBDE concentration among the six POPs, with a value of 109 ± 115 ng/g d.w. with the range of 0.291(<MDL)-313 ng/g d.w. (R: 0.291(<MDL)-313) (Table S16). The mean values of Σ_17_PCDD/Fs and Σ_12_PBDD/Fs were 241 ± 131 (R: 0.634 (<MDL)-476) and 227 ± 216 (R: 18.2(<MDL)-654), respectively, while the average TEQ concentrations of Σ_17_PCDD/Fs and Σ_12_PBDD/Fs were 2.59 ± 1.11(R: 0.111(<MDL)-3.46) and 1.04 ± 0.307 (R: 0.535(<MDL)-1.49) pg WHO_2005_-TEQ/g d.w., respectively (Tables S11 and S14). The detectable Σ_12_DL-PCB concentrations ranged from 3.03 (<MDL) to 494 (mean ± SD: 102 ± 160) pg/g d.w., exhibiting a more than 10-fold change (Table S12). In addition, their concentrations varied between the sampling locations and times. At a depth of 2000 m, Σ_14_PBDE concentrations ranged from 0.291 (<MDL) to 313 ng/g dry weight (d.w.). The highest Σ_14_PBDE concentrations were observed in sample A from September to November 2013. At this sampling depth, there was a sampling section from January to early February in 2014 (sample C) where the concentrations of six POPs were all lower than the MDLs. At a depth of 3500 m, Σ_14_PBDE concentrations ranged from 21.5 to 236 ng/g d.w., which also had the highest mean concentration among the six POPs, with a value of 96.1 ± 95.3 ng/g d.w. at the same depth. Similar to the POP levels at 2000 m, Σ_17_PCDD/F and Σ_12_PBDD/F concentrations at a water depth of 3500 m were 237 ± 32.5 (R: 214–285) (2.90 ± 0.438 (R: 2.48–3.46) pg WHO_2005_-TEQ/g d.w.) and 219 ± 153 (R: 85.1–439) pg/g d.w. (1.13 ± 0.293 (R: 0.811–1.49) pg WHO_2005_-TEQ/g d.w.), respectively. Notably, the mean concentration of Σ_12_DL-PCBs (54.2 ± 11.1 (R: 45.6–70.0) pg/g d.w.) was relatively low at 3500 m. At the water depths of 2000 m and 3500 m, PCDEs were not detectable (<MDLs) and most of the PBB compounds were present below the MDLs; only a few samples (B, G, and H) were detected in low concentrations, ranging from 5.79 to 11.2 pg/g d.w. Apart from PCDEs and PBBs, the mean POP concentrations at a depth of 3500 m were generally lower than those at 2000 m. A similar trend was also found in WHO_2005_-TEQs of chlorinated and brominated dioxins. However, there were no significant differences among POPs compounds (*p* > 0.05). The compositions of PCDD/Fs, DL-PCBs, PBDD/Fs, PBBs, and PBDEs are shown in Figure 3. Similar POP patterns were present in the pooled A, B, D, E, F, G, and H samples.

In Table 2, the highest mean concentration of PCDD/F compounds was that of OCDD at depths of 3500 m and 2000 m, with mean concentrations of (R: 140–221) and 165 ± 123 (R: 0.0418(<MDL)-290) pg/g d.w., respectively. PBDD concentrations were lower than the MDLs in all of the deposited particulate samples. Only a few PBDF congeners, including OctBDF and 1,2,3,4,6,7,8-HpBDF, were observed (200 ± 269 (R: 12.9(<MDL)-590) and 29.9 ± 26.2 (R: 0.917(<MDL)-64.1) pg/g d.w.) at a depth of 2000 m, and in similar concentrations at 3500 m (OctBDF: 166 ± 127 (R: 58.6–349) pg/g d.w. and 1,2,3,4,6,7,8-HpBDF: 48.3 ± 27.0 (R: 21.5–85.9) pg/g d.w.). PCB-126 concentrations, which had the highest TEF of DL-PCBs, ranged from 0.197 (<LOD) to 4.32 pg/g d.w. (mean ± SD: 1.62 ± 1.84 and 1.03 ± 0.169 pg/g d.w. in 2000 and 3500 m, respectively) in all the analyzed samples. Octa-BDE, Nona-BDE, and Deca-BDE levels were several times greater in samples A and E than in other samples. The mean concentrations of Octa-BDEs, Nona-BDEs, and Deca-BDEs were 0.767 ± 0.608 (R: 0.0161 (<MDL)-1.47), 19.6 ± 27.1 (R:0.0526 (<MDL)-58.4), and 101 ± 119 (R: 0.166 (<MDL)-253) ng/g d.w., respectively, at a depth of 2000 m and 0.877 ± 0.740 (R: 0.313–1.97), 12.3 ± 12.6 (R: 2.72–8.10), and 82.2 ± 81.5 (R: 17.9–202) ng/g d.w. at a depth of 3500 m, respectively.

**Figure 4:**
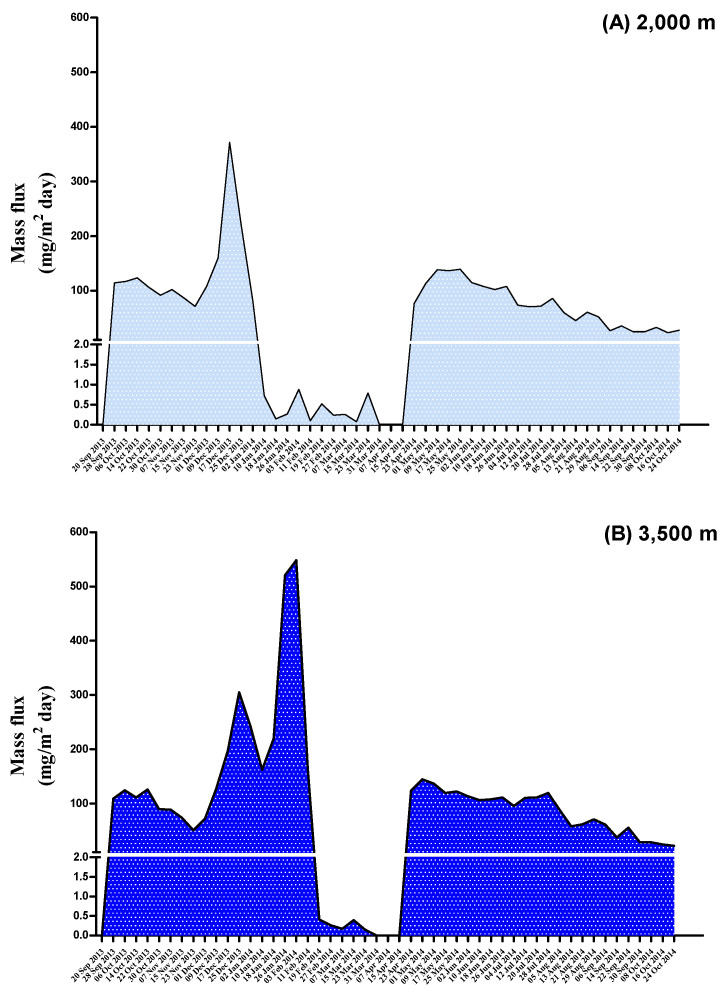
The time trend for the mass flux of deposited particulate at the depths of 2000 and 3500 m between 20 September 2013 and 24 October 2014.

The time trends for the mass flux of deposited particulates between September 2013 and October 2014 are shown in Figure 4. As shown in Table 3, the mass flux of deposited particulates ranged from 0.8 to 189 mg/m^2^/day at a depth of 2000 m, while it ranged from 89.3 to 323 mg/m^2^/day at a depth of 3500 m. The total volume of particles in sample C is negligible (0.800 mg/m^2^/day). If only considering POP compounds, PBDEs (depths of 2000 m and 3500 m: 20.9 and 14.2 ng/m^2^/day, respectively) had the highest mass flux of the deposited particulates, followed by PCDD/Fs (36.4 and 39.9 pg/m^2^/day, respectively), PBDD/Fs (36.5 and 34.6 pg/m^2^/day, respectively), and DL-PCBs (21.1 and 9.08 pg/m^2^/day, respectively). WHO_2005_-TEQ levels for the mass flux of deposited particulates at a depth of 2000 m were 0.339, 0.0257, and 0.126 pg-WHO_2005_-TEQ/m^2^/day for PCDD/Fs, DL-PCBs, and PBDD/Fs, respectively, while their values at a depth of 3500 m were 0.528, 0.0218, and 0.200 pg-WHO_2005_-TEQ/m^2^/day, respectively. The mass flux of deposited particulates for PBBs and PCDEs was not calculated due to most of the measurements of these chemicals being below the LOD. The mass fluxes of PCDD/Fs, DL-PCBs, PBDD/Fs, and PBDEs in the whole year were calculated as 119, 68.7, 119, and 68,200 pg/m^2^/year at a depth of 2000 m, respectively, as well as 132, 76.5, 132, and 75,900 pg/m^2^/year at the depth of 3500 m, respectively. For the dioxin-like compounds, mass fluxes of PCDD/F-TEQs, DL-PCB-TEQs, and PBDD/F-TEQs of 1.10, 0.0839, and 0.410 pg-WHO_2005_-TEQ/m^2^/year, respectively, at 2000 m were lower than those whose TEQ values of 1.23, 0.0933, and 0.457 pg-WHO_2005_-TEQ/m^2^/year, respectively, at 3500 m.

## 4. Discussion

Among the six studied POPs, PBDEs are the predominant species in deposited particulates in the marine environment according to our findings. PBDE levels, particularly BDE-209, in the deposited particulates are significantly higher than the other POPs in the SCS. The marine environment plays an important role in the fate of BFRs through aerosol deposition, land runoffs, and river discharges, which further adds to the accumulation of particulates in oceanic sediments [74,75]. Many previous studies have also noted that BDE-209 is the dominant PBDE congener in the marine environment [68,74,76], particularly in sediments from rivers [77], lakes [78], and seas [68]. Deca-BDE has a lower toxicity than other commercial PBDE mixtures such as Penta- and Octa-BDEs; thus, its component BDE-209 is more commonly detected in the marine environment, especially in aquatic media, due to large amounts of BDE-209 in various matrices [79]. In this study, commercial Octa-BDE constituents such as BDE-206, -207, and -208 were also detected in the deposited particulate samples, which highlights the possibility that commercial Octa-BDEs are contaminating marine resources in the SCS, even though it has been banned. However, the possibility that explains the high occurrence of Octa- and Nona-BDEs is BDE-209 debromination via sunlight in the aquatic environment, which is highly likely in the river sediments, as determined in previous studies [77,80,81,82] to be further transported to the ocean. LRAT plays a major role in PBDE congener air transport and deposition, especially from mainland China, where BDE-209 use is prevalent and where it is a dominant congener in the environment [83].

Several scientists have calculated marine deposit fluxes from the atmosphere, including gaseous, dry, and wet deposition, estimating a dry deposition of 0.005 ng/m^2^/day for BFRs (of which the major contributor is BDE-47) in the eastern Atlantic and the Southern Ocean [84]; a gaseous deposition of 1 ng/m^2^/day for BDE-47, BDE-99, and BDE-209, as well as dry deposition of 0.05, 0.01, and 0.1 ng/m^2^/day, respectively, in the tropical and subtropical Atlantic Ocean [85]; and a dry deposition of up to about 0.1 ng/m^2^/day for BDE-47 and BDE-99 in the Arctic and 2.9 ng/m^2^/day on the east Asian Pacific coast [86]. A Japanese research team calculated the surficial flux of BDE-209 and PBDEs (from Di-BDEs to Nona-BDEs) in sediments collected from Tokyo Bay to be 509–1700 (13,900–46,600 ng/m^2^/day) and 17–58 (466–1590 ng/m^2^/day) ng/cm^2^/year, respectively [87]. Few research works have studied deposited particulates in the ocean after PBDE transport from land to the marine environment. Our PBDE mass flux for marine deposition in the deep-sea SCS was notably higher than atmospheric deposition mass fluxes from the other marine studies, but still obviously lower than the Japanese report on the surficial flux in the sediment of Tokyo Bay.

For dioxin or dioxin-like compounds in the aquatic environment, increasing chlorination increases the hydrophobicity of the compound. Based on our results, OCDD and OcBDF are the main constituents of PCDD/Fs and PBDD/Fs, respectively, and PCB-105 and -118 are the main constituents of DL-PCBs in marine-deposited particulates. In previous studies [65,88,89,90,91,92,93], dioxin-like compounds were found in sea water and sea sediments in open sea areas. If only dioxin-like compounds are considered, in the present study, the PBDF levels were the highest in the deposited particulates, followed by PCDD, DL-PCB, and PCDF, in that order. This is inconsistent with a Japanese study, which determined that in marine sediments the concentration decreases in the order: PCDDs, DL-PCBs, PCDFs, PBDFs, and PBDDs [65]. We determined PCDD/F and DL-PCB mass fluxes of 0.339 and 0.0257 pg-WHO_2005_-TEQ/m^2^/day, respectively, at a depth of 2000 m, and 0.528 and 0.0218 pg-WHO_2005_-TEQ/m^2^/day, respectively, at 3500 m for the deposited particulates (Table 3). Korhonen et al. (2013) [91] determined sedimentation rates of 20–59 pg-WHO-TEQ/m^2^/day for PCDD/Fs and 0.5–3.2 pg-WHO-TEQ/m^2^/day for DL-PCBs in the open sea of the Gulf of Finland and the Gulf of Bothnia. In our report (Table S11), the TEQs of 1,2,3,7,8-PeCDD (0.508 ± 3.56 pg-WHO_2005_-TEQ/g d.w.) and 2,3,4,7,8-PeCDF (0.510 ± 3.57 pg-WHO_2005_-TEQ/g d.w.) were the major contributors to the total WHO_2005_-TEQ for the deposited particulates. This was consistent with a previous study that revealed that these congeners dominate sediments deposited in the western Gulf of Finland, the Gulf of Bothnia, and the Archipelago Sea [90]. Atmospheric PCDD/F dry deposition fluxes of 234 and 5–170 pg/m^2^/day were determined in the Black and Mediterranean Seas, respectively [88]. This article also indicated that PCDD/Fs in the open Mediterranean Sea are possibly transported by LRAT from the continent and across the Atlantic and also introduced via shipping emissions. Furthermore, considering all the contributing mechanisms, including diffusive air/water fluxes, air/water fugitive ratios, the net volatilization, and the net absorption flux, atmospheric deposition processes were finally recognized as the net PCDD/F sink in the open Mediterranean Sea [88]. Jurado et al. (2004) [90] showed that aerosol deposition fluxes to the open sea, including dry deposition and air–water exchange, are mainly influenced by the wind speed, temperature, and sinking fluxes. Particles in the marine environment are deposited by both atmospheric deposition and sedimentation. Few reports have detailed the marine POP deposition in open seas.

Currently, there is a lack of data on PBBs or PCDEs in marine environments. PBB congeners, i.e., BB-15 and BB-194, were found in certain deposited particulates in the SCS in the present study. The PBB congener BB-153 was detected in sediments collected from the mouth of the Saginaw River by Yun et al. (2008) [94]. According to the mechanisms and reactions of PBBs, BB-194, which was detected in high amounts in deposited particulates, is theorized to have been produced from BB-209 via photodebromination. However, due to a lack of data, it is impossible to determine how significant this photochemical reaction is regarding the degradation or transformation of PBBs in the atmosphere (ATSDR 2004). In this study, the PCDE levels in marine-deposited particulates were lower than the MDLs. PCDEs have been discovered in numerous environmental matrices in China, including surface sediments, SPM, and water [95,96]. Although, in our study, PCDE congeners were detected below the MDLs, their degradation resistance and biological toxicity require additional consideration. In addition, our results might be influenced by internal waves in the SCS, which is one of the seas with the largest reported internal waves in the world [97,98]. Therefore, due to this disruption (Figure S3), POP mass fluxes in the deep-sea particulate were presented the diversity and large variation in the present study.

POP presence in deep-ocean-deposited particulates in the SCS possibly originates from atmospheric and aquatic environments, especially from nearby continents. POPs in the marine environment have historically been determined to be present at the global background levels. Although it is difficult to identify the persistence and atmospheric transport behavior of halogenated POPs in the environment, scientific models have shown that these halogenated compounds can move to the oceanic environment via LRAT [50,89,98]. Our previous study established the atmospheric background levels of PCDD/Fs, PBDD/Fs, DL-PCBs, PBBs, PCDEs, and PBDEs over the Pacific Ocean near to southern Taiwan and the northern Philippines [2], adding to the limited existing atmospheric global data. PCBs and PBDEs were the most dominant halogenated compounds in the global oceanic atmosphere, in contrast to previous studies. Aside from LRAT, POPs can be transported to the SCS via global distillation/fractionation effects, as the SCS has a strong surface current system, with warm water running through the Pacific Ocean and the Indian Ocean [69]. The SCS is also affected by a tropical marine monsoon climate with high temperatures and humidities [99]. The historic atmospheric PCDD/F level at Dongsha Island located in the northern SCS, close to our sampling site (SEATS), was found to be 1.52–10.8 fg I-TEQ/m^3^ (n = 17), which was attributed to LRAT after an Asian Dust Storm (ADS) event originating from mainland China [100]. Atmospheric PBDE concentrations were observed in the northern SCS in relation to the continental PBDEs outflows from Pearl River Delta (PRD) on the southeast coasts of mainland China, the Philippines, and Taiwan by LART, particularly for northeast wind transport [101]. Aside from atmospheric samples, sediment samples are also essential for POP risk evaluation. Mai et al. (2005) [77] evaluated the spatial and temporal distributions of PBDEs in 66 surface sediment samples from the Pearl River Delta (PRD) and the adjacent SCS and observed BDE-209 and PBDE (nine congeners excluding BDE-209) concentrations ranging from 0.4 to 7340 and from 0.04 to 94.7 ng/g d.w., respectively. Bioaccumulation of POPs such as PCBs, PBDEs, and PCDD/Fs has already been observed in SCS marine organisms such as fish [67,102,103,104]; marine mammals, such as dolphins and porpoises [105,106]; crabs [107]; whales [15]; octopuses [107]; and striated cones [107]. The POPs in the SCS oceanic atmosphere and sediments and PRD sediments indirectly reflect the POPs in SCS deep-ocean-deposited particulates detected in the present study, which further explains their source. POPs in water reservoirs in South China might indirectly support emission sources from the land (Table S16). In previous environmental research focused on the SCS, several studies reported POP contamination in surface water, in the atmosphere, in sediments, and in aquatic biota [15,100,101,102,107]. To summarize, based on previous reports and the present study, the POPs in deposited particulates may have come from marine surface water, river discharges, reverse liquid phase transit, or atmospheric LART.

## 5. Conclusions

This study is the first to establish an investigation of POPs in marine-deposited particulate samples from the SCS. First, PCDD/F, DL-PCB, PBDE, PBDD/F, PCDE, and PBB background levels were determined in SCS deep-ocean-deposited particulates. PBDEs, particularly BDE-209, were the major constituents of deposited particles in the marine environment, followed by PCDD/Fs, PBDD/Fs, and PCBs. POPs such as PCDD/Fs, DL-PCBs, and PBDD/Fs were also detected in the deposited particulates in the present study. Most PBB and PCDE levels were under the LODs. Of the 66 POP compounds, BDE-209 was found to be a significant and notable contaminant of deposited particles in the SCS marine environment. PBDE mass fluxes in the deposited particulates at a depth of 2000 m were higher than those at a depth of 3500 m, but the differences were statistically insignificant. Ultimately, the ocean is regarded as one of the final POP sinks. Our findings in the current investigation show that POP pollutants are present in deposited particles in the SCS maritime environment. This investigation may provide an understanding of the environmental fate of these POPs after surface-sinking and before sedimentation in the ocean.

## Figures and Tables

**Table 1:** Levels of PCDD/Fs, PCBs, PCDEs, PBDD/Fs, PBBs, and PBDEs in the deposited particulates collected from two deep-ocean sites (2000 and 3500 m).

Deep-Ocean Site	Σ_17_PCDD/Fs (pg/g d.w.)	Σ_17_PCDD/Fs (pg WHO_2005_-TEQ/g d.w.)	Σ_12_DL-PCBs (pg/g d.w.)	Σ_12_PCBs (pg WHO_2005_-TEQ/g d.w.)	Σ_6_PCDEs (pg/g d.w.)	Σ_12_PBDD/Fs (pg/g d.w.)	Σ_12_PBDD/Fs (pg WHO_2005_-TEQ/g d.w.)	Σ_5_ PBBs (pg/g d.w.)	Σ_14_PBDEs (ng/g d.w.)
**Location: SEAT 1 (17°59.685′ N, 116°00.1408′ E)**									
**Sailing Period 2013/09/20 ~ 2013/11/23)**									
A (2000 m)	476	3.39	494	0.471	5.37 ^a^	654	1.34	5.73 ^a^	313
E (3500 m)	285	3.03	70.0	0.134	5.37 ^a^	439	1.49	5.73 ^a^	236
**Sailing Period (2013/11/23 ~ 2014/01/02)**									
B (2000 m)	208	2.24	46.8	0.103	5.37 ^a^	205	0.890	11.2	157
F (3500 m)	230	2.48	54.0	0.129	5.37 ^a^	189	0.993	5.73 ^a^	66.4
**Sailing Period (2014/01/02 ~ 2014/03/23)**									
C (2000 m)	0.634 ^a^	0.100 ^a^	3.03 ^a^	0.000303 ^a^	5.37 ^a^	18.2 ^a^	0.536 ^a^	5.73 ^a^	0.291 ^a^
G (3500 m)	214	3.46	47.5	0.129	5.37 ^a^	164	1.23	6.54	60.7
**Location: SEAT 2 (18°06.3852′ N, 116°01.9695′ E)**									
**Sailing Period (2014/04/15 ~ 2014/10/24)**									
D (2000 m)	294	3.39	56.2	0.134	5.37 ^a^	57.8	1.00	5.73 ^a^	17.8
H (3500 m)	220	2.64	45.6	0.0947	5.37 ^a^	85.1	0.811	5.79	21.5
2000 m Mean ± SD	245 ± 197	2.28 ± 1.55	150 ± 230	0.177 ± 0.204	5.37 ^a^	234 ± 291	0.94 ± 0.332	7.11	122 ± 145
3500 m Mean ± SD	237 ± 32.5	2.90 ± 0.438	54.2 ± 11.1	0.122 ± 0.0181	5.37 ^a^	219 ± 153	1.13 ± 0.293	5.95	96.1 ± 95.3
*p*-value ^b^	1.00	1.00	0.715	0.715	-	1.00	0.465	1.00	0.465
*p*-value ^c^	1.00	0.686	1.00	0.886	-	0.886	0.686	0.886	0.886

^a^ Measurements below the MDL were set as 1/2 the MDL. ^b^ Wilcoxon test (2000 m vs. 3500 m). ^c^ Mann–Whitney *U* Test (2000 m vs. 3500 m).

**Table 2:** Levels of certain POPs on the deposited particulate collected in two deep-ocean sites of 2000 and 3500s m ^a^.

	Depths of 2000 m					Depths of 3500 m				
	A	B	C	D	Mean ± SD	E	F	G	H	Mean ± SD
**PCDD/Fs (pg/g d.w.)**										
2,3,7,8-TeCDD	0.171	0.170	0.0387 ^a^	0.150	0.132 ± 0.0632	0.220	0.169	0.154	0.135	0.170 ± 0.0364
1,2,3,7,8-PeCDD	0.583	0.376	0.0339 ^a^	0.563	0.389 ± 0.254	0.599	0.472	0.511	0.454	0.509 ± 0.0645
OCDD	290	155	0.0418 ^a^	213	165 ± 123	221	176	140	163	175 ± 34.1
**PCBs (pg/g d.w.)**										
PCB-126	4.32	0.849	0.197 ^a^	1.12	1.62 ± 1.84	1.14	1.12	1.08	0.779	1.03 ± 0.169
**PBDD/Fs (pg/g d.w.)**										
1,2,3,4,6,7,8-HpBDF	64.1	31.7	0.917 ^a^	22.7	29.9 ± 26.2	85.9	42.8	42.8	21.5	48.3 ± 27.0
OctBDF	590	169	12.9 ^a^	28.2	200 ± 269	349	142	115	58.6	166 ± 127
**PBDEs **										
Tri-BDE (pg/g d.w.)	5.50	5.23	1.91 ^a^	6.88	4.88 ± 2.11	5.58	2.13	3.35	4.87	3.98 ± 1.55
Tetra-BDE (pg/g d.w.)	63.4	55.1	12.8 ^a^	74.6	51.5 ± 27.0	63.2	51.8	39.2	46.6	50.2 ± 10.1
Penta-BDE (pg/g d.w.)	61.3	27.1	21.1 ^a^	70.1	44.9 ± 24.4	64.5	30.2	36.5	36.5	41.9 ± 15.3
Hexa-BDEs (pg/g d.w.)	85.2	94.0	8.38 ^a^	394	145 ± 170	297	131	119	144	173 ± 83.5
Hepta-BDEs (pg/g d.w.)	240	257	12.6 ^a^	1300	452 ± 576	1040	356	330	348	517 ± 345
Octa-BDEs (pg/g d.w.)	1470	635	16.1 ^a^	949	767 ± 608	1970	662	567	313	877 ± 740
Nona-BDEs (ng/g d.w.)	58.4	17.9	0.0526 ^a^	2.06	19.6 ± 27.1	30.9	8.10	7.42	2.72	12.3 ± 12.7
Deca-BDE was (ng/g d.w.)	253	138	0.166 ^a^	13.0	101 ± 119	202	57.1	52.2	17.9	82.2 ± 81.5

**Table 3:** Mass flux of PCDD/Fs, PCBs, PCDEs, PBDD/Fs, PBBs, and PBDEs in deposited particulates collected from two deep-ocean sites (2000 and 3500 m).

	Depths of 2000 m					Depths of 3500 m				
	A	B	C	D	Mean ± SD	E	F	G	H	Mean ± SD
**Mass flux (mg/m^2^/day)**	102	189	0.8	73.3	91.3 ± 77.8	96.5	188	323	89.3	174 ± 109
**PCDD/Fs (pg/m^2^/day)**	48.5	39.2	–	21.5	36.4 ± 13.7	27.5	43.3	69.1	19.6	39.9 ± 21.8
2,3,7,8-TeCDD	0.0174	0.0321	–	0.0110	0.0202 ± 0.0108	0.0212	0.0318	0.0497	0.0121	0.0287 ± 0.0162
1,2,3,7,8-PeCDD	0.0594	0.0709	–	0.0123	0.0572 ± 0.0149	0.0578	0.0888	0.165	0.0405	0.0880 ± 0.0551
OCDD PCDD/F-TEQs ^a^	29.5 0.345	29.2 0.422	– –	15.6 0.248	24.8 ± 7.96 0.339 ± 0.0872	21.3 0.292	33.1 0.467	45.2 1.12	14.6 0.236	28.6 ± 13.5 0.528 ± 0.405
**DL-PCBs (pg/m^2^/day)**	50.3	8.83	-	4.12	21.1 ± 25.4	6.75	10.2	15.3	4.07	9.08 ± 4.86
PCB-126 PCB-TEQs ^a^	0.440 0.0480	0.160 0.0194	– –	0.0821 0.00982	0.227 ± 0.188 0.0257 ± 0.0199	0.110 0.0129	0.211 0.0243	0.349 0.0417	0.0696 0.00846	0.185 ± 0.124 0.0218 ± 0.0148
**PBDD/Fs (pg/m^2^/day)**	66.6	38.7	–	4.24	36.5 ± 31.3	42.4	35.6	53.0	7.60	34.6 ± 19.4
1,2,3,4,6,7,8-HpBDF	6.53	5.98	–	1.66	4.72 ± 2.66	8.29	8.06	13.8	1.92	8.02 ± 4.86
OctBDF PBDD/F-TEQs ^a^	60.1 0.137	31.9 0.168	– –	2.07 0.0734	31.4 ± 29.0 0.126 ± 0.0481	33.7 0.143	26.7 0.187	37.1 0.396	5.23 0.0724	25.7 ± 14.3 0.200 ± 0.139
**PBDEs (pg/m^2^/day)**	31,929	29,561	–	1305	20,932 ± 17,038	22,756	12,496	19,593	1920	14,191 ± 9237
Tri-BDEs	0.561	0.986	–	0.504	0.684 ± 0.264	0.538	0.400	1.083	0.435	0.614 ± 0.318
Tetra-BDEs	6.46	10.4	–	5.46	7.44 ± 2.60	6.10	9.75	12.6	4.16	8.16 ± 3.78
Penta-BDEs	6.25	5.12	–	5.14	5.51 ± 0.644	6.23	5.70	11.8	3.26	6.75 ± 3.61
Hexa-BDEs	8.69	17.7	–	28.9	18.5 ± 10.1	28.7	24.7	38.6	12.9	26.2 ± 10.6
Hepta-BDEs	24.5	48.5	–	95.0	56.0 ± 35.9	99.8	67.1	107	31.1	76.1 ± 34.7
Octa-BDEs	150	120	–	69.6	113 ± 40.5	190	125	183	27.9	131 ± 74.9
Nona-BDEs	5945	3375	–	151	3157 ± 2903	2981	1524	2395	243	1786 ± 1190
Deca-BDEs	25,787	25,984	–	950	17,574 ± 14,397	19,444	10,740	16,845	1598	12,156 ± 7928

^a^ The units of the TEQ are pg-WHO_2005_-TEQ/m^2^/day.

## Data Availability

The data presented in this study are available on request from the corresponding author.

## Supplementary Materials

The following supporting information can be downloaded at https://www.mdpi.com/article/10.3390/toxics11120968/s1: Table S1: GC columns and temperature conditions of GC oven used in the present study; Table S2: The recovery rate (%) of spiked PCDD/F, PCB, and PCDE standards; Table S3: The recovery rate (%) of spiked PBDD/F, PBB, and PBDE standards; Table S4: The recovery rate of PCDD/F standards in the deposited particulate; Table S5: The recovery rate of PBDD/F standards in the deposited particulate; Table S6: The recovery rate of PCB standards in the deposited particulate; Table S7: The recovery rate of PBDE standards in the deposited particulate; Table S8: Levels of PCDD/Fs, PCBs, and PCDEs in blank; Table S9: Levels of PBDD/Fs, PBBs, and PBDEs in blank; Table S10: Method detection limits (MDLs) of PBBs, PBDD/Fs, PBDEs, DL-PCBs, PCDD/Fs, and PCDEs (pg/g d.w.); Table S11: Concentrations of PCDD/Fs in the deposited particulate (pg/g d.w.); Table S12: Concentrations of DL-PCBs in the deposited particulate (pg/g d.w.); Table S13: Concentrations of PCDEs in the deposited particulate (pg/g d.w.); Table S14: Concentrations of PBDD/Fs in the deposited particulate (pg/g d.w.); Table S15: Concentrations of PBBs in the deposited particulate (pg/g d.w.); Table S16: Concentrations of PBDEs in the deposited particulate (pg/g d.w.).; Table S17: POP levels in water reservoirs of South China; Figure S1: (a) The anchorage-type sediment trap (b) The two-series anchorage-type sediment trap; Figure S2: The analytical procedure of POPs in the present study; Figure S3: Sample C is collection of deposited particulate between 2 January 2014 and 11 February 2014. Little mass is obtained at the depth of 2000 m during this duration, possibly due to internal wave condition [71,108,109,110,111,112,113,114].
