# Supplementary material for: Persistent Halogenated Organic Pollutants in Deep-Water-Deposited Particulates from South China Sea
Source: Toxics. 2023 Nov 29;11(12):968. doi: 10.3390/toxics11120968 (PMC10748163; doi:10.3390/toxics11120968)
Supplement: Supplementary file 1 [file toxics-11-00968-s001.zip › toxics-2643157-Supplemental Materials.pdf]

Supplemental Materials

Persistent Halogenated Organic Pollutants in Deep Water Deposited Particulate from South China Sea

Jia-De Lee[1+], Tsyr-Huei Chiou[2+], Hong-Jie Zhang[3], How-Ran Chao [1,4,5,6,7*], Kuang-Yu Chen [8+], Yan-You Gou[1], Chien-Er Huang[9,10], Sheng-Lun Lin[11], Lin-Chi Wang [12]**

[1] Department of Environmental Science and Engineering, National Pingtung University of Science and Technology, Neipu, Pingtung County 91201, Taiwan; jdlee01251212@gmail.com (J-D L); hrchao@mail.npust.edu.tw (H-R C); power20342000@gmail.com (Y-Y G)

[2] Department of Life Sciences, National Cheng Kung University, Tainan City 701, Taiwan; thchiou@mail.ncku.edu.tw

[3] School of Mechanical Engineering, Beijing Institute of Technology, Beijing, China; 3220210320@bit.edu.cn

[4] Center for Agricultural, Forestry, Fishery, Livestock and Aquaculture Carbon Emission Inventory and Emerging Compounds, General Research Service Center, National Pingtung University of Science and Technology, Neipu, Pingtung 91201, Taiwan

[5] Institute of Food Safety Management, College of Agriculture, National Pingtung University of Science and Technology, Pingtung 91201, Taiwan

[6] School of Dentistry, College of Dental Medicine, Kaohsiung Medical University, Kaohsiung City 807, Taiwan

[7] Department of Occupational Safety and Health, Faculty of Public Health, Universitas Airlangga, Kampus C Mulyorejo Surabaya 60115, Indonesia.

[8] Taiwan Ocean Research Institute, National Applied Research Laboratories, Kaohsiung City 852, Taiwan; littlefish3@short.idv.tw

[9] Department of Mechanical Engineering/ Institute of Mechanical Engineering, Cheng Shiu University, Niaosong District, Kaohsiung City 833, Taiwan; k1668@gcloud.csu.edu.tw

[10] Super Micro Mass Research & Technology Center, Cheng Shiu University, Niaosong District, Kaohsiung City 833, Taiwan

[11] Department of Environmental Engineering, National Cheng Kung University, Tainan City 701, Taiwan; z1120811@ncku.edu.tw

[12] Department of Marine Environmental Engineering, National Kaohsiung University of Science and Technology, Nanzih District, Kaohsiung City 81157, Taiwan; lcwang@nkust.edu.tw

+ These authors equally contributed.

* Corresponding author: Prof. How-Ran Chao (Ph.D.)
E-mail: hrchao@mail.npust.edu.tw
TEL: +886-87703202 ext. 7517; FAX: +886-87740256
Address: Department of Environmental Science and Engineering, National Pingtung University and Science and Technology, 1 Shuefu Road, Neipu Township, Pingtung County 912, Taiwan

** Additional corresponding author: Prof. Lin-Chi Wang (Ph.D.)
E-mail: lcwang@ nkust.edu.tw
Address: Department of Marine Environmental Engineering, National Kaohsiung University of Science and Technology, No.142, Haijhuan Road, Nanzih District, Kaohsiung City 81157, Taiwan
Tel: +886-7-3617141; Fax:+886-7-3649259.

Tables and Figures

Tables

Table S1 GC columns and temperature conditions of GC oven used in the present study

Table S2 The recovery rates (%) of spiked PCDD/F, DL-PCB, and PCDE standards

Table S3 The recovery rates (%) of spiked PBDD/F, PBB, and PBDE standards

Table S4 The recovery rates of PCDD/F standards in the deposited particulate

Table S5 The recovery rates of PBDD/F standards in the deposited particulate

Table S6 The recovery rates of DL-PCB standards in the deposited particulate

Table S7 The recovery rates of PBDE standards in the deposited particulate

Table S8 Levels of PCDD/Fs, DL-PCBs, and PCDEs in blank

Table S9 Levels of PBDD/Fs, PBBs, and PBDEs in blank

Table S10 Method detection limits (MDLs) of PBBs, PBDD/Fs, PBDEs, DL-PCBs, PCDD/Fs, and PCDEs (pg/g d.w.)

Table S11 Concentrations of PCDD/Fs in the deposited particulate (pg/g d.w.)

Table S12 Concentrations of DL-PCBs in the deposited particulate (pg/g d.w.)

Table S13 Concentrations of PCDEs in the deposited particulate (pg/g d.w.)

Table S14 Concentrations of PBDD/Fs in the deposited particulate (pg/g d.w.)

Table S15 Concentrations of PBBs in the deposited particulate (pg/g d.w.)

Table S16 Concentrations of PBDEs in the deposited particulate (pg/g d.w.)

Table S17 POP levels in water reservoirs of South China

Figures

Figure S1. (a) The anchorage-type sediment trap (b) The two-series anchorage-type sediment trap

Figure S2 The analytical procedure of POPs in the present study

Figure S3. Sample C is collection of the deposited particulate between 2014/1/2 and 2014/2/11. Little mass is obtained at the depth of 2000 m during this duration possibly due to internal wave condition.

Table S1 GC columns and temperature conditions of GC oven used in the present study

Compound	GC column	Injector temperature	Temperature program
PCDD/Fs	Column: DB-5MS, 60 m, 0.25 mm i.d., 0.25 µm film thickness (J&W Scientific, CA)	290 ºC	began at 150 ºC (held for 1 min), 30 ºC/min to 220 ºC (held for 12 min), then increased at 1.5 ºC/min to 240 ºC (held for 5 min), and finally increased at 1.5 ºC/min to 310 ºC (held for 20 min).
PCBs	Column: DB-5MS, 60 m, 0.25 mm i.d., 0.25 µm film thickness (J&W Scientific, CA)	290 ºC	began at 140 ºC, then increased at 20 ºC/min to 210 ºC, then increased at 1.5 ºC/min to 240 ºC, and finally increased at 20 ºC/min to 310 ºC (held for 4 min)
PBDD/Fs	Column: Rtx-5MS, 30 m, 0.25 mm i.d., 0.25 µm film thickness (Restek, PA)	290 ºC	began at 150 ºC (held for 1 min), then increase at 40 ºC/min to 220 ºC, then increase at 2 ºC /min to 240 ºC, and finally increase at 10 ºC/min to 310 ºC (held for 1 min).
PBBs	Column: Rtx-5MS, 30 m, 0.25 mm i.d., 0.25 µm film thickness (Restek, PA)	270 ºC	began at 100 ºC (held for 4 min), then increased at 30 ºC/min to 160 ºC (held for 2 min), and finally increased at 10 ºC/min to 315 ºC (held for 2 min)
PBDEs	Column: DB-5HT, 15 m, 0.25 mm i.d., 0.1 µm film thickness (J&W Scientific, CA)	250 ºC	began at 100 °C (held for 4 min), then increased at 40 °C/min to 200 °C (held for 3.5 min), and finally increased at 10 °C/min to 325 °C (held for 2.5 min).

Table S2 The recovery rates (%) of spiked PCDD/F, DL-PCB, and PCDE standards

PCDD/Fs	Recovery rate of PCDD/Fs (%)	Criteria of recovery rate (%)	PCBs	Recovery rate of PCBs (%)	Criteria of recovery rate (%)	PCDEs	Recovery rate of PCDEs (%)	Criteria of recovery rate (%)
2,3,7,8-TeCDF	94.46	70% ~ 130%	PCB#77(4CL)	111.43	60%-140%	CDE#28(3CL)	115.2	20~130
1,2,3,7,8-PeCDF	95.39	70% ~ 130%	PCB#81(4CL)	113.20	60%-140%	CDE#77(4CL)	111.3	30~130
2,3,4,7,8-PeCDF	96.01	70% ~ 130%	PCB#105(5CL)	121.32	60%-140%	CDE#99(5CL)	109.4	60~130
1,2,3,4,7,8-HxCDF	97.92	70% ~ 130%	PCB#114(5CL)	115.15	60%-140%	CDE#141(6CL)	128.4	60~130
1,2,3,6,7,8-HxCDF	94.08	70% ~ 130%	PCB#118(5CL)	122.72	60%-140%	CDE#180(7CL)	118.2	60~130
2,3,4,6,7,8-HxCDF	98.30	70% ~ 130%	PCB#123(5CL)	116.84	60%-140%	CDE#209(10CL)	64.0	60~200
1,2,3,7,8,9-HxCDF	100.72	70% ~ 130%	PCB#126(5CL)	119.49	60%-140%			
1,2,3,4,6,7,8-HpCDF	102.66	70% ~ 130%	PCB#156(6CL)	107.00	60%-140%			
1,2,3,4,7,8,9-HpCDF	101.24	70% ~ 130%	PCB#157(6CL)	102.60	60%-140%			
OCDF	109.60	70% ~ 130%	PCB#167(6CL)	114.07	60%-140%			
2,3,7,8-TeCDD	97.13	70% ~ 130%	PCB#169(6CL)	107.43	60%-140%			
1,2,3,7,8-PeCDD	94.40	70% ~ 130%	PCB#189(7CL)	90.54	60%-140%			
1,2,3,4,7,8-HxCDD	92.29	70% ~ 130%						
1,2,3,6,7,8-HxCDD	100.10	70% ~ 130%						
1,2,3,7,8,9-HxCDD	95.47	70% ~ 130%						
1,2,3,4,6,7,8-HpCDD	97.43	70% ~ 130%						
OCDD	100.46	70% ~ 130%						

Table S3 The recovery rates (%) of spiked PBDD/F, PBB, and PBDE standards

PBDD/Fs	Recovery rate of PBDD/Fs (%)	Criteria of recovery rate (%)	PBBs	Recovery rate of PBBs (%)	Criteria of recovery rate (%)	PBDEs	Recovery rate of PBDEs (%)	Criteria of recovery rate (%)
2,3,7,8-TeBDF	121.30	60% ~ 140%	PBB#15(2Br)	103.1	50%-150%	BDE #28(3Br)	98.1	60~140%
1,2,3,7,8-PeBDF	99.88	60% ~ 140%	PBB#52(4Br)	126.7	60%-140%	BDE #47(4Br)	105.2	60~140%
2,3,4,7,8-PeBDF	100.11	60% ~ 140%	PBB#153(6Br)	129.2	60%-140%	BDE #100(5Br)	105.8	60~140%
1,2,3,4,7,8-HxBDF	98.30	60% ~ 140%	PBB#180(7Br)	90.9	60%-140%	BDE #99(5Br)	100.1	60~140%
1,2,3,4,6,7,8-HpBDF	104.72	60% ~ 140%	PBB#194(8Br)	121.6	60%-140%	BDE #154(6Br)	98.6	60~140%
OctBDF	131.22	50% ~ 150%				BDE #153(6Br)	95.1	60~140%
2,3,7,8,-TeBDD	119.49	60% ~ 140%				BDE #183(7Br)	97.3	60~140%
1,2,3,7,8-PeBDD	98.70	60% ~ 140%				BDE #197(8Br)	99.6	60~140%
1,2,3,4/6,7,8-HxBDD	96.24	60% ~ 140%				BDE #203(8Br)	103.5	60~140%
1,2,3,7,8,9-HxBDD	105.25	60% ~ 140%				BDE #196(8Br)	109.0	60~140%
1,2,3,4,6,7,8-HpBDD	92.08	60% ~ 140%				BDE #208(9Br)	103.7	60~140%
OctBDD	95.95	50% ~ 150%				BDE #207(9Br)	97.3	60~140%
						BDE #206(9Br)	78.9	60~140%
						BDE #209(10Br)	112.8	60~140%

Table S4 The recovery rates of PCDD/F standards in the deposited particulate

	Deposited particulate							BK[a]			Criteria of recovery rate (%)
	A	B	C	D	E	F	G	Filter BK	Store BK	Sea BK	
Internal Standards											
$^{13}C_{12}$-2,3,7,8-TeCDF	95.2	84.6	91.8	91.8	101.9	93.5	89.4	77.4	92.9	97.1	30%~130%
$^{13}C_{12}$-1,2,3,7,8-PeCDF	93.7	86.6	93.6	94.7	101.6	97.9	92.2	88.8	105.4	105.2	30%~130%
$^{13}C_{12}$-2,3,4,7,8-PeCDF	100.0	89.9	98.0	94.9	105.6	99.4	95.7	79.7	97.5	99.6	30%~130%
$^{13}C_{12}$-1,2,3,4,7,8-HxCDF	102.0	93.8	100.2	98.9	111.2	103.7	95.6	91.6	109.4	107.3	40%~130%
$^{13}C_{12}$-1,2,3,6,7,8-HxCDF	101.3	94.4	100.8	101.0	112.0	104.0	96.2	92.3	110.9	110.2	40%~130%
$^{13}C_{12}$-2,3,4,6,7,8-HxCDF	105.0	95.7	102.1	101.1	111.5	104.9	97.8	83.0	101.5	101.0	40%~130%
$^{13}C_{12}$-1,2,3,7,8,9-HxCDF	107.5	94.1	104.3	99.8	111.2	104.7	97.9	81.6	101.2	98.6	40%~130%
$^{13}C_{12}$-1,2,3,4,6,7,8-HpCDF	103.0	93.5	100.3	100.8	107.3	101.9	94.6	86.4	107.2	104.0	40%~130%
$^{13}C_{12}$-1,2,3,4,7,8,9-HpCDF	103.2	93.8	102.4	97.8	107.9	99.8	95.2	81.1	97.4	98.9	40%~130%
$^{13}C_{12}$-2,3,7,8-TeCDD	87.8	81.3	90.1	90.2	95.7	93.5	88.7	76.6	95.7	97.5	30%~130%
$^{13}C_{12}$-1,2,3,7,8-PeCDD	95.2	91.3	98.5	96.2	103.6	100.0	95.4	81.1	101.5	101.7	30%~130%
$^{13}C_{12}$-1,2,3,4,7,8-HxCDD	101.5	94.4	102.4	99.2	110.2	105.0	97.9	83.5	104.3	103.3	40%~130%
$^{13}C_{12}$-1,2,3,6,7,8-HxCDD	100.3	91.3	99.9	101.1	108.2	103.4	96.2	82.1	102.7	101.7	40%~130%
$^{13}C_{12}$-1,2,3,4,6,7,8-HpCDD	100.1	91.9	101.2	99.9	107.0	99.3	95.5	75.5	99.0	99.2	40%~130%
$^{13}C_{12}$-OCDD	87.5	80.9	90.7	86.0	93.8	86.7	84.4	59.3	82.9	84.4	40%~130%
Cleanup standard											
$^{37}C_{14}$-2,3,7,8-TeCDD	86.8	80.6	89.6	90.1	94.1	92.4	88.1	71.9	89.7	92.2	30%~130%

[a] BK means blank, Filter BK means filter blank, Store BK means store blank, and Sea BK means sea blank.

Table S5 The recovery rates of PBDD/F standards in the deposited particulate

	Deposited particulate							BK			Criteria of recovery rate (%)
	A	B	C	D	E	F	G	Filter BK	Store BK	Sea BK	
Internal standards											
L-13C12-2,3,7,8-TeBDF	64.9	64.7	83.1	72.5	66.1	71.1	73.9	73.0	28.4	45.1	20%~150%
L-13C12-1,2,3,7,8-PeBDF	50.8	58.7	53.2	48.4	53.9	49.8	56.6	57.1	24.9	35.2	20%~150%
L-13C12-2,3,4,7,8-PeBDF	54.5	57.0	56.3	50.0	54.5	49.3	56.0	53.2	26.0	37.6	20%~150%
L-13C12-1,2,3,4,7,8-HxBDF	65.4	54.5	54.6	56.6	61.1	56.9	59.6	72.1	39.6	58.5	20%~150%
L-13C12-1,2,3,4,6,7,8-HpBDF	75.1	75.2	84.2	71.9	85.7	86.2	101.1	137.1	130.6	144.0	20%~150%
L-13C12-OctBDF	47.0	54.7	59.7	38.7	49.8	50.9	60.6	13.0	12.8	12.0	10%~150%
L-13C12-2,3,7,8-TeBDD	57.5	52.6	58.7	48.2	49.8	51.0	60.9	58.7	22.9	36.3	20%~150%
L-13C12-1,2,3,7,8-PeBDD	54.7	54.7	58.7	55.1	59.4	53.1	66.0	80.8	38.5	50.5	20%~150%
L-13C12-1,2,3,4/6,7,8-HxBDD	55.3	61.8	63.0	58.0	66.1	63.3	64.9	93.9	55.9	76.2	20%~150%
L-13C12-1,2,3,7,8,9-HxBDD	82.8	63.9	71.6	64.8	71.3	60.4	70.7	109.0	65.4	85.2	20%~150%
L-13C12-1,2,3,4,6,7,8-HpBDD	60.0	64.5	70.0	62.8	66.7	73.6	90.3	98.8	88.4	107.6	20%~150%
L-13C12-OctBDD	59.9	69.0	76.6	62.8	72.1	78.7	85.3	35.8	36.8	45.7	10%~150%

Table S6 The recovery rates of DL-PCB standards in the deposited particulate

	Deposited particulate							BK			Criteria of recovery rate (%)
	A	B	C	D	E	F	G	Filter BK	Store BK	Sea BK	
Internal Standards											
L-PCB#77(4CL)	87.6	82.0	82.8	86.5	89.3	88.1	96.1	78.3	88.9	100.4	25% ~ 150%
L-PCB#81(4CL)	82.9	77.4	80.2	82.5	86.4	83.8	90.4	74.3	83.7	92.7	25% ~ 150%
L-PCB#105(5CL)	30.5	63.9	29.3	49.7	51.5	28.8	53.5	62.6	45.2	58.1	25% ~ 150%
L-PCB#114(5CL)	34.7	35.5	25.1	30.8	36.4	27.1	26.3	33.4	30.0	25.2	25% ~ 150%
L-PCB#118(5CL)	26.6	44.4	25.8	34.2	36.7	33.8	41.8	50.8	30.4	41.6	25% ~ 150%
L-PCB#123(5CL)	29.1	33.2	28.4	26.1	27.8	27.6	32.9	43.0	27.3	32.6	25% ~ 150%
L-PCB#126(5CL)	88.8	144.2	88.9	109.4	91.8	87.3	92.1	67.6	79.7	86.0	25% ~ 150%
L-PCB#156(6CL)	31.3	27.2	54.8	45.5	40.9	54.1	44.0	50.2	31.9	44.9	25% ~ 150%
L-PCB#157(6CL)	43.1	38.2	31.6	62.2	54.5	31.0	56.5	61.4	43.8	58.4	25% ~ 150%
L-PCB#167(6CL)	46.4	39.2	59.3	40.5	36.7	35.3	41.3	50.6	31.9	41.2	25% ~ 150%
L-PCB#169(6CL)	126.8	90.4	104.5	139.2	105.2	98.3	101.3	70.0	83.4	93.5	25% ~ 150%
L-PCB#189(7CL)	35.5	39.5	31.0	32.0	35.4	38.1	40.5	55.9	34.7	48.3	25% ~ 150%

Table S7 The recovery rates of PBDE standards in the deposited particulate

	Deposited particulate							BK			Criteria of recovery rate (%)
	A	B	C	D	E	F	G	Filter BK	Store BK	Sea BK	
Internal Standards											
L-BDE #28 (3Br)	35.9	36.5	45.5	9.5	71.6	47.8	48.8	58.3	70.8	58.2	20~150%
L-BDE #47 (4Br)	45.2	55.4	65.0	11.4	111.3	66.7	69.0	70.1	86.0	70.6	20~150%
L-BDE #99 (5Br)	37.6	48.6	55.5	10.4	94.4	64.0	63.7	70.7	88.0	76.7	20~150%
L-BDE #154 (6Br)	35.6	30.8	47.5	8.8	107.1	47.9	44.0	67.8	75.0	62.6	20~150%
L-BDE #153 (6Br)	36.5	45.3	55.0	8.7	102.5	57.6	61.3	63.5	78.8	64.7	20~150%
L-BDE #183 (7Br)	39.6	34.4	53.3	9.6	102.5	57.9	50.4	63.6	79.4	68.0	20~150%
L-BDE #197 (8Br)	42.7	51.7	71.5	10.6	103.4	78.5	75.4	63.2	81.6	78.8	20~150%
L-BDE #207 (9Br)	44.8	52.4	62.7	10.4	114.5	78.6	68.6	73.2	82.6	72.3	20~150%
L-BDE #209 (10Br)	90.8	87.1	85.6	14.4	164.6	114.1	94.0	100.5	112.6	86.5	20~150%

Table S8 Levels of PCDD/Fs, PCBs, and PCDEs in blank

	Compound	Filter BK	Store BK	Sea BK	1040820BK-6	Mean	SD	Max	Min
PCDD/Fs	2,3,7,8-TeCDF	ND	ND	ND	ND	ND	ND	ND	ND
	1,2,3,7,8-PeCDF	ND	ND	ND	ND	ND	ND	ND	ND
	2,3,4,7,8-PeCDF	ND	ND	ND	ND	ND	ND	ND	ND
	1,2,3,4,7,8-HxCDF	ND	ND	ND	ND	ND	ND	ND	ND
	1,2,3,6,7,8-HxCDF	ND	ND	ND	ND	ND	ND	ND	ND
	2,3,4,6,7,8-HxCDF	ND	ND	ND	ND	ND	ND	ND	ND
	1,2,3,7,8,9-HxCDF	ND	ND	ND	ND	ND	ND	ND	ND
	1,2,3,4,6,7,8-HpCDF	0.147	ND	0.219	ND	0.0915	0.110	0.219	ND
	1,2,3,4,7,8,9-HpCDF	ND	ND	ND	ND	ND	ND	ND	ND
	OCDF	0.395	2.28	0.703	0.229	0.902	0.940	2.28	0.229
	2,3,7,8-TeCDD	ND	ND	ND	ND	ND	ND	ND	ND
	1,2,3,7,8-PeCDD	ND	ND	ND	ND	ND	ND	ND	ND
	1,2,3,4,7,8-HxCDD	ND	ND	ND	ND	ND	ND	ND	ND
	1,2,3,6,7,8-HxCDD	ND	ND	ND	ND	ND	ND	ND	ND
	1,2,3,7,8,9-HxCDD	ND	ND	ND	ND	ND	ND	ND	ND
	1,2,3,4,6,7,8-HpCDD	0.236	ND	ND	ND	0.0590	0.118	0.236	ND
	OCDD	1.23	1.22	1.44	0.366	1.06	0.476	1.44	0.366
	Σ_{17} PCDD/Fs (pg)	2.01	3.50	2.36	0.595	2.11	1.64	4.18	0.595
PCBs	PCB-77(4CL)	2.20	2.19	2.05	0.299	1.68	0.926	2.20	0.299
	PCB-81(4CL)	ND	ND	ND	ND	ND	ND	ND	ND
	PCB-105(5CL)	1.87	2.22	1.68	0.713	1.62	0.645	2.22	0.713
	PCB-114(5CL)	ND	ND	ND	ND	ND	ND	ND	ND
	PCB-118(5CL)	3.73	4.07	3.96	1.55	3.33	1.19	4.07	1.55
	PCB-123(5CL)	ND	ND	ND	ND	ND	ND	ND	ND
	PCB-126(5CL)	ND	ND	ND	ND	ND	ND	ND	ND
	PCB-156(6CL)	ND	ND	ND	ND	ND	ND	ND	ND
	PCB-157(6CL)	ND	ND	ND	ND	ND	ND	ND	ND
	PCB-167(6CL)	ND	ND	ND	ND	ND	ND	ND	ND
	PCB-169(6CL)	ND	ND	ND	ND	ND	ND	ND	ND
	PCB-189(7CL)	ND	ND	ND	ND	ND	ND	ND	ND
	Σ_{12} PCBs (pg)	7.80	8.48	7.69	2.56	6.63	2.76	8.49	2.56
PCDEs	CDE-28(3CL)	ND	ND	ND	ND	ND	ND	ND	ND
	CDE-77(4CL)	ND	ND	ND	ND	ND	ND	ND	ND
	CDE-99(5CL)	ND	ND	ND	ND	ND	ND	ND	ND
	CDE-141(6CL)	ND	ND	ND	ND	ND	ND	ND	ND
	CDE-180(7CL)	ND	ND	ND	ND	ND	ND	ND	ND
	CDE-209(10CL)	ND	ND	ND	ND	ND	ND	ND	ND
	Σ_6 PCDEs (pg)	ND	ND	ND	ND	ND	ND	ND	ND

Table S9 Levels of PBDD/Fs, PBBs, and PBDEs in blank

	Compound	Filter BK	Store BK	Sea BK	1040820BK-6	Mean	SD	Max	Min
PBDD/Fs	2,3,7,8-TeBDF	ND	ND	ND	ND	ND	ND	ND	ND
	1,2,3,7,8-PeBDF	ND	ND	ND	ND	ND	ND	ND	ND
	2,3,4,7,8-PeBDF	ND	ND	ND	ND	ND	ND	ND	ND
	1,2,3,4,7,8-HxBDF	2.17	1.53	ND	1.19	1.22	0.911	2.17	ND
	1,2,3,4,6,7,8-HpBDF	3.85	2.23	ND	ND	1.52	1.88	3.85	ND
	OctBDF	29.8	ND	ND	ND	7.45	14.9	29.8	ND
	2,3,7,8,-TeBDD	ND	ND	ND	ND	ND	ND	ND	ND
	1,2,3,7,8-PeBDD	ND	ND	ND	ND	ND	ND	ND	ND
	1,2,3,4/6,7,8-HxBDD	ND	ND	ND	ND	ND	ND	ND	ND
	1,2,3,7,8,9-HxBDD	ND	ND	ND	ND	ND	ND	ND	ND
	1,2,3,4,6,7,8-HpBDD	ND	ND	ND	ND	ND	ND	ND	ND
	OctBDD	ND	ND	ND	ND	ND	ND	ND	ND
	Σ_{12} PBDD/Fs (pg)	35.8	3.76	ND	1.19	10.2	17.7	35.8	ND
PBBs	PBB-15(2Br)	ND	ND	ND	ND	ND	ND	ND	ND
	PBB-52(4Br)	ND	ND	ND	ND	ND	ND	ND	ND
	PBB-153(6Br)	ND	ND	ND	ND	ND	ND	ND	ND
	PBB-180(7Br)	ND	ND	ND	ND	ND	ND	ND	ND
	PBB-194(8Br)	ND	ND	ND	ND	ND	ND	ND	ND
	Σ_5 PBBs (pg)	ND	ND	ND	ND	ND	ND	ND	ND
PBDEs	BDE-28(3Br)	1.92	1.92	1.92	1.92	1.92	0.000	1.92	1.92
	BDE-47(4Br)	12.8	12.8	12.8	12.8	12.8	0.000	12.8	12.8
	BDE-100(5Br)	2.27	2.27	2.27	2.27	2.27	0.000	2.27	2.27
	BDE-99(5Br)	18.5	18.5	18.5	18.5	18.5	0.000	18.5	18.5
	BDE-154(6Br)	2.13	2.13	2.13	2.13	2.13	0.000	2.13	2.13
	BDE-153(6Br)	6.25	6.25	6.25	6.25	6.25	0.000	6.25	6.25
	BDE-183(7Br)	12.6	42.5	12.6	12.6	20.1	15.0	42.5	12.6
	BDE-197(8Br)	21.6	40.6	15.2	7.02	21.1	14.3	40.6	7.02
	BDE-203(8Br)	42.1	41.4	18.0	5.32	26.7	18.1	42.1	5.32
	BDE-196(8Br)	41.9	45.3	19.2	3.76	27.5	19.6	45.3	3.76
	BDE-208(9Br)	51.7	46.2	63.2	43.1	51.1	8.85	63.2	43.1
	BDE-207(9Br)	96.7	88.7	93.2	79.0	89.4	7.67	96.7	79
	BDE-206(9Br)	100	73.7	35.4	15.6	56.2	37.9	100	15.6
	BDE-209(10Br)	1863	1462	732	921	1245	515	1863	732
	Σ_{14} PBDEs (pg)	2273	1884	1033	1131	1580	637	2337	942

Table S10 Method detection limits (MDLs) of PBBs, PBDD/Fs, PBDEs, DL-PCBs, PCDD/Fs, and PCDEs (pg/g d.w.)

Bromination			Chlorination		
**PBBs**	MDL	1/2 MDL	**DL-PCBs**	MDL	1/2 MDL
PBB#15(2Br)	1.27	0.634	PCB#77(4CL)	0.366	0.183
PBB#52(4Br)	0.0730	0.0365	PCB#81(4CL)	0.138	0.0690
PBB#153(6Br)	8.36	4.18	PCB#105(5CL)	1.000	0.500
PBB#180(7Br)	1.32	0.658	PCB#114(5CL)	0.530	0.265
PBB#194(8Br)	0.448	0.224	PCB#118(5CL)	1.23	0.615
**PBDD/Fs**			PCB#123(5CL)	0.213	0.107
2,3,7,8-TeBDF	0.0700	0.0350	PCB#126(5CL)	0.393	0.197
1,2,3,7,8-PeBDF	0.174	0.0870	PCB#156(6CL)	0.557	0.279
2,3,4,7,8-PeBDF	0.264	0.132	PCB#157(6CL)	0.399	0.200
1,2,3,4,7,8-HxBDF	0.618	0.309	PCB#167(6CL)	0.802	0.401
1,2,3,4,6,7,8-HpBDF	1.83	0.917	PCB#169(6CL)	0.133	0.0666
OctBDF	25.7	12.9	PCB#189(7CL)	0.301	0.150
2,3,7,8,-TeBDD	0.0470	0.0235	**PCDD/Fs**		
1,2,3,7,8-PeBDD	0.537	0.269	2,3,7,8-TeCDF	0.0798	0.0399
1,2,3,4/6,7,8-HxBDD	2.54	1.27	1,2,3,7,8-PeCDF	0.0576	0.0288
1,2,3,7,8,9-HxBDD	0.322	0.161	2,3,4,7,8-PeCDF	0.0562	0.0281
1,2,3,4,6,7,8-HpBDD	2.04	1.02	1,2,3,4,7,8- HxCDF	0.0542	0.0271
OctBDD	2.18	1.09	1,2,3,6,7,8-HxCDF	0.0543	0.0271
**PBDEs**			2,3,4,6,7,8-HxCDF	0.0541	0.0271
BDE #28(3Br)	3.83	1.91	1,2,3,7,8,9-HxCDF	0.0570	0.0285
BDE #47(4Br)	25.6	12.8	1,2,3,4,6,7,8-HpCDF	0.0637	0.0318
BDE #100(5Br)	4.54	2.27	1,2,3,4,7,8,9-HpCDF	0.0801	0.0401
BDE #99(5Br)	37.0	18.5	OCDF	0.0966	0.0483
BDE #154(6Br)	4.26	2.13	2,3,7,8-TeCDD	0.0775	0.0387
BDE #153(6Br)	12.5	6.25	1,2,3,7,8-PeCDD	0.0679	0.0339
BDE #183(7Br)	25.1	12.6	1,2,3,4,7,8-HxCDD	0.0797	0.0398
BDE #197(8Br)	14.0	7.02	1,2,3,6,7,8-HxCDD	0.0836	0.0418
BDE #203(8Br)	10.6	5.32	1,2,3,7,8,9-HxCDD	0.0800	0.0400
BDE #196(8Br)	7.51	3.76	1,2,3,4,6,7,8-HpCDD	0.130	0.0649
BDE #208(9Br)	28.0	13.98	OCDD	0.0970	0.0485
BDE #207(9Br)	46.0	23.0	**PCDEs**		
BDE #206(9Br)	31.3	15.6	CDE#28(3CL)	6.93	3.47
BDE #209(10Br)	333	166	CDE#77(4CL)	0.371	0.185
			CDE#99(5CL)	0.311	0.156
			CDE#141(6CL)	0.778	0.389
			CDE#180(7CL)	0.396	0.198
			CDE#209(10CL)	1.94	0.968

Table S11 Concentrations of PCDD/Fs in the deposited particulate (pg/g d.w.)

	A	B	C	D	E	F	G	H	Mean	SD	Max	Min
2,3,7,8-TeCDD	0.171	0.170	0.0387 [b]	0.150	0.220	0.169	0.154	0.135	0.151	0.0517	0.220	0.0387
1,2,3,7,8-PeCDD	0.583	0.376	0.0339 [b]	0.563	0.599	0.472	0.511	0.454	0.449	0.183	0.599	0.0339
1,2,3,4,7,8-HxCDD	0.677	0.434	0.0398 [b]	0.667	0.675	0.547	0.546	0.521	0.513	0.210	0.677	0.0398
1,2,3,6,7,8-HxCDD	1.44	0.714	0.0418 [b]	1.11	1.11	0.841	0.998	0.868	0.890	0.408	1.44	0.0418
1,2,3,7,8,9-HxCDD	1.80	1.05	0.0400 [b]	1.67	1.71	1.32	1.32	1.26	1.27	0.561	1.80	0.0400
1,2,3,4,6,7,8-HpCDD	27.1	12.1	0.0649 [b]	18.4	16.9	13.7	13.0	13.9	14.4	7.53	27.1	0.0649
OCDD	290	155	0.0485 [b]	213	221	176	140	163	170	83.6	290	0.0485
2,3,7,8-TeCDF	0.986	0.666	0.0399 [b]	0.950	0.983	0.783	0.996	0.802	0.776	0.321	1.00	0.0399
1,2,3,7,8-PeCDF	1.64	1.17	0.0288 [b]	1.88	1.52	1.34	2.20	1.45	1.40	0.642	2.20	0.0288
2,3,4,7,8-PeCDF	1.77	1.41	0.0281 [b]	1.95	1.58	1.39	2.26	1.53	1.49	0.660	2.26	0.0281
1,2,3,4,7,8-HxCDF	3.01	2.19	0.0271 [b]	3.85	2.77	2.40	4.42	2.94	2.70	1.31	4.42	0.0271
1,2,3,6,7,8-HxCDF	2.84	2.15	0.0271 [b]	3.64	2.81	2.26	4.09	2.75	2.57	1.22	4.09	0.0271
2,3,4,6,7,8-HxCDF	2.86	2.06	0.0271 [b]	3.57	2.66	2.24	3.92	2.73	2.51	1.18	3.92	0.0271
1,2,3,7,8,9-HxCDF	0.293	0.266	0.0285 [b]	0.346	0.377	0.262	0.340	0.229	0.268	0.109	0.377	0.0285
1,2,3,4,6,7,8-HpCDF	25.3	9.9	0.0318 [b]	17.8	12.0	10.5	18.7	13.0	13.4	7.47	25.3	0.0318
1,2,3,4,7,8,9-HpCDF	2.23	1.24	0.0401 [b]	2.14	1.82	1.41	2.35	1.62	1.61	0.746	2.35	0.0401
OCDF	113	16.7	0.0483 [b]	21.5	16.8	14.3	18.6	12.9	26.7	35.4	113	0.0483
Σ_{17} PCDD/Fs	476	208	0.634	294	285	230	214	220	241	131	476	0.634
Σ_{17} PCDD/F-TEQ [a]	3.39	2.24	0.100	3.39	3.03	2.48	3.46	2.64	2.59	1.11	3.46	0.111

[a] pg WHO_{2005}-TEQ/g

[b] it means the concentration lower than MDL and it is expressed as <1/2 MDL.

Table S12 Concentrations of DL-PCBs in the deposited particulate (pg/g d.w.)

	A	B	C	D	E	F	G	H	Mean	SD	Max	Min
PCB-77(4CL)	50.7	5.19	0.183 [b]	9.87	7.98	6.32	5.23	6.95	11.6	16.1	50.7	0.183
PCB-81(4CL)	1.29	0.0690 [b]	0.0690 [b]	0.559	0.0690 [b]	0.0690 [b]	0.367	0.296	0.349	0.422	1.29	0.0690
PCB-105(5CL)	182	11.1	0.500 [b]	13.3	16.1	12.1	11.1	9.61	32.0	60.8	182	0.500
PCB-114(5CL)	9.31	0.265 [b]	0.265 [b]	0.265	0.265 [b]	0.265 [b]	0.265 [b]	0.84	1.47	3.18	9.31	0.265
PCB-118(5CL)	133	21.3	0.615 [b]	22.4	34.5	24.9	21.9	21.1	35.0	40.7	133	0.615
PCB-123(5CL)	3.98	0.107 [b]	0.107 [b]	2.34	0.107 [b]	0.955	0.107 [b]	0.107 [b]	0.976	1.45	3.98	0.107
PCB-126(5CL)	4.32	0.849	0.197 [b]	1.12	1.14	1.12	1.08	0.779	1.33	1.25	4.32	0.197
PCB-156(6CL)	71.5	3.08	0.279 [b]	2.09	4.71	3.98	2.81	2.80	11.4	24.3	71.5	0.279
PCB-157(6CL)	15.7	1.07	0.200 [b]	1.34	1.29	1.11	1.15	0.911	2.85	5.21	15.7	0.200
PCB-167(6CL)	17.1	2.36	0.401 [b]	1.30	2.33	1.96	1.62	1.25	3.54	5.52	17.1	0.401
PCB-169(6CL)	0.672	0.553	0.0666 [b]	0.664	0.577	0.494	0.630	0.497	0.519	0.195	0.672	0.666
PCB-189(7CL)	4.11	0.897	0.150 [b]	1.03	0.896	0.778	1.29	0.540	1.21	1.22	4.11	0.150
Σ_{12} DL-PCBs	494	46.8	3.03	56.2	70	54	47.5	45.6	102	160	494	3.03
Σ_{12} DL-PCB-TEQ [a]	0.471	0.103	0.000303	0.134	0.134	0.129	0.129	0.0947	0.149	0.137	0.471	0.000303

[a] pg WHO_{2005}-TEQ/g

[b] it means the concentration lower than MDL and it is expressed as <1/2 MDL.

Table S13 Concentrations of PCDEs in the deposited particulate (pg/g d.w.)

	A	B	C	D	E	F	G	H	Mean	SD	Max	Min
PCDE-28(3CL)	3.47 [a]	3.47 [a]	3.47 [a]	3.47 [a]	3.47 [a]	3.47 [a]	3.47 [a]	3.47 [a]	3.47 [a]	0.00	3.47	3.47
PCDE-77(4CL)	0.185 [a]	0.185 [a]	0.185 [a]	0.185 [a]	0.185 [a]	0.185 [a]	0.185 [a]	0.185 [a]	0.185 [a]	0.00	0.185	0.185
PCDE-99(5CL)	0.156 [a]	0.156 [a]	0.156 [a]	0.156 [a]	0.156 [a]	0.156 [a]	0.156 [a]	0.156 [a]	0.156 [a]	0.00	0.156	0.156
PCDE-141(6CL)	0.389 [a]	0.389 [a]	0.389 [a]	0.389 [a]	0.389 [a]	0.389 [a]	0.389 [a]	0.389 [a]	0.389 [a]	0.00	0.389	0.389
PCDE-180(7CL)	0.198 [a]	0.198 [a]	0.198 [a]	0.198 [a]	0.198 [a]	0.198 [a]	0.198 [a]	0.198 [a]	0.198 [a]	0.00	0.198	0.198
PCDE-209(10CL)	0.968 [a]	0.968 [a]	0.968 [a]	0.968 [a]	0.968 [a]	0.968 [a]	0.968 [a]	0.968 [a]	0.968 [a]	0.00	0.968	0.968
Σ_6 PCDEs	5.37	5.37	5.37	5.37	5.37	5.37	5.37	5.37	5.37	0.00	5.37	5.37

[a] it means the concentration lower than MDL and it is expressed as <1/2 MDL.

Table S14 Concentrations of PBDD/Fs in the deposited particulate (pg/g d.w.)

	A	B	C	D	E	F	G	H	Mean	SD	Max	Min
2,3,7,8,-TeBDD	0.0235 [b]	0.0235 [b]	0.0235 [b]	0.0235 [b]	0.0235 [b]	0.0235 [b]	0.0235 [b]	0.0235 [b]	0.0235	0.00	0.0235	0.0235
1,2,3,7,8-PeBDD	0.269 [b]	0.269 [b]	0.269 [b]	0.269 [b]	0.269 [b]	0.269 [b]	0.269 [b]	0.269 [b]	0.269	0.00	0.269	0.269
1,2,3,4/6,7,8-HxBDD	1.27 [b]	1.27 [b]	1.27 [b]	1.27 [b]	1.27 [b]	1.27 [b]	1.27 [b]	1.27 [b]	1.27	0.00	1.27	1.27
1,2,3,7,8,9-HxBDD	0.161 [b]	0.161 [b]	0.161 [b]	0.161 [b]	0.161 [b]	0.161 [b]	0.161 [b]	0.161 [b]	0.161	0.00	0.161	0.161
1,2,3,4,6,7,8-HpBDD	1.02 [b]	1.02 [b]	1.02 [b]	1.02 [b]	1.02 [b]	1.02 [b]	1.02 [b]	1.02 [b]	1.02	0.00	1.02	1.02
OctBDD	1.09 [b]	1.09 [b]	1.09 [b]	1.09 [b]	1.09 [b]	1.09 [b]	1.09 [b]	1.09 [b]	1.09	0.00	1.09	1.09
2,3,7,8-TeBDF	0.035 [b]	0.035 [b]	0.035 [b]	0.035 [b]	0.035 [b]	0.035 [b]	0.035 [b]	0.035 [b]	0.035	0.00	0.035	0.035
1,2,3,7,8-PeBDF	0.087 [b]	0.087 [b]	0.087 [b]	0.087 [b]	0.087 [b]	0.087 [b]	0.087 [b]	0.087 [b]	0.087	0.00	0.087	0.087
2,3,4,7,8-PeBDF	0.132 [b]	0.132 [b]	0.132 [b]	0.132 [b]	0.132 [b]	0.132 [b]	0.132 [b]	0.132 [b]	0.132	0.00	0.132	0.132
1,2,3,4,7,8-HxBDF	0.309 [b]	0.309 [b]	0.309 [b]	2.74	0.309 [b]	0.309 [b]	2.72	0.870	0.984	1.09	2.74	0.309
1,2,3,4,6,7,8-HpBDF	64.1	31.7	0.917 [b]	22.7	85.9	42.8	42.8	21.5	39.1	26.5	85.9	0.917
OctBDF	590	169	12.9 [b]	28.2	349	142	115	58.6	183	196	590	12.9
Σ_{12} PBDD/Fs	654	205	18.2	57.8	439	189	164	85.1	227	216	654	18.2
Σ_{12} PBDD/F-TEQ [a]	1.34	0.890	0.536	1.00	1.49	0.993	1.23	0.811	1.04	0.307	1.49	0.535

[a] pg WHO_{2005}-TEQ/g

[b] it means the concentration lower than MDL and it is expressed as <1/2 MDL.

Table S15 Concentrations of PBBs in the deposited particulate (pg/g d.w.)

	A	B	C	D	E	F	G	H	Mean	SD	Max	Min
PBB-15(2Br)	0.634 [a]	0.634 [a]	0.634 [a]	0.634 [a]	0.634 [a]	0.634 [a]	1.45	0.689	0.743	0.286	1.45	0.634
PBB-52(4Br)	0.0365 [a]	0.0365 [a]	0.0365 [a]	0.0365 [a]	0.0365 [a]	0.0365 [a]	0.0365 [a]	0.0365 [a]	0.0365	0.00	0.0365	0.0365
PBB-153(6Br)	4.18 [a]	4.18 [a]	4.18 [a]	4.18 [a]	4.18 [a]	4.18 [a]	4.18 [a]	4.18 [a]	4.18	0.00	4.18	4.18
PBB-180(7Br)	0.658 [a]	0.658 [a]	0.658 [a]	0.658 [a]	0.658 [a]	0.658 [a]	0.658 [a]	0.658 [a]	0.658	0.00	0.658	0.658
PBB-194(8Br)	0.224 [a]	5.73	0.224 [a]	0.224 [a]	0.224 [a]	0.224 [a]	0.224 [a]	0.224 [a]	0.912	1.95	5.73	0.224
Σ_5 PBBs	5.73	11.2	5.73	5.73	5.73	5.73	6.54	5.79	6.53	1.92	11.2	5.73

[a] it means the concentration lower than MDL and it is expressed as <1/2 MDL.

Table S16 Concentrations of PBDEs in the deposited particulate (pg/g d.w.)

	A	B	C	D	E	F	G	H	Mean	SD	Max	Min
BDE-28(3Br)	5.50	5.23	1.91 [a]	6.88	5.58	2.13	3.35	4.87	4.43	1.78	6.88	1.91
BDE-47(4Br)	63.4	55.1	12.8 [a]	74.6	63.2	51.8	39.2	46.6	50.8	18.9	74.6	12.8
BDE-100(5Br)	12.8	7.30	2.27 [a]	11.9	10.3	9.77	7.02	5.75	8.39	3.48	12.8	2.27
BDE-99(5Br)	48.5	19.9	18.5 [a]	58.3	54.2	20.5	29.6	30.8	35.0	16.3	58.3	18.5
BDE-154(6Br)	28.8	26.9	2.13 [a]	88.4	67.3	36.1	33.5	39.4	40.3	26.4	88.4	2.13
BDE-153(6Br)	56.5	67.2	6.25 [a]	306	230	95.0	85.9	105	119	98.9	306	6.25
BDE-183(7Br)	240	257	12.6 [a]	1297	1035	356	330	348	484	440	1297	12.6
BDE-197(8Br)	363	204	7.02 [a]	526	733	206	206	130	297	234	733	7.02
BDE-203(8Br)	535	210	5.32 [a]	153	626	234	181	85.3	254	216	626	5.32
BDE-196(8Br)	572	221	3.76 [a]	270	606	222	180	97.6	272	213	606	3.76
BDE-208(9Br)	11238	3224	13.98 [a]	395	7366	1691	1479	528	3242	4006	11238	14.0
BDE-207(9Br)	22168	6462	23.0 [a]	1003	12775	3194	2831	1052	6189	7656	22168	23.0
BDE-206(9Br)	24944	8214	15.6 [a]	660	10757	3213	3106	1139	6506	8353	24944	15.6
BDE-209(10Br)	253076	137791	166 [a]	12962	201510	57055	52171	17892	91578	94878	253076	166
Σ_{14}PBDEs	313352	156765	291	17812	235840	66386	60683	21504	109079	114685	313352	291

[a] it means the concentration lower than MDL and it is expressed as <1/2 MDL.

Table S17 POP levels in water reservoirs of South China

Country	Sampling year	Concentrations						References
		PCDD/Fs	PCBs	PCDEs	PBDD/Fs	PBBs	PBDEs	
**Water** (ng/L)								
Pearl River Estuary	2000		33.38-1064					[109]
South China Sea	2000		21.72-144					[110]
Tributaries of a large city in South China.	2017						0.70-13.12 [a] 0.46-2.33 [b]	[111]
Beijiang River	2014						<LOD – 0.232	[112]
Pearl River Delta	2005-2006						0.34-68.0	[113]
Hong Kong's coastal waters	2005						0.0113-0.228	[114]
Xijiang River,	2005-2006	0.00265-0.00459						[115]

a: Dry season b: Wet season

The memory of RV Ocean Researcher V (海研五號)

RV Ocean Researcher V, which was a research vessel, owned by the Taiwan Ocean Research Institute, Ministry of Science and Technology, Taiwan, and this research ship was sank off the coast of Penghu, Taiwan in October 10, 2014. Our laboratory and our team joined the first and final sail of RV Ocean Researcher V to collect atmospheric persistent organic pollutants over the Pacific Ocean near south Taiwan and north Phillipine and to gather the persistent organic pollutants on deep water deposited particulate in South China Sea.

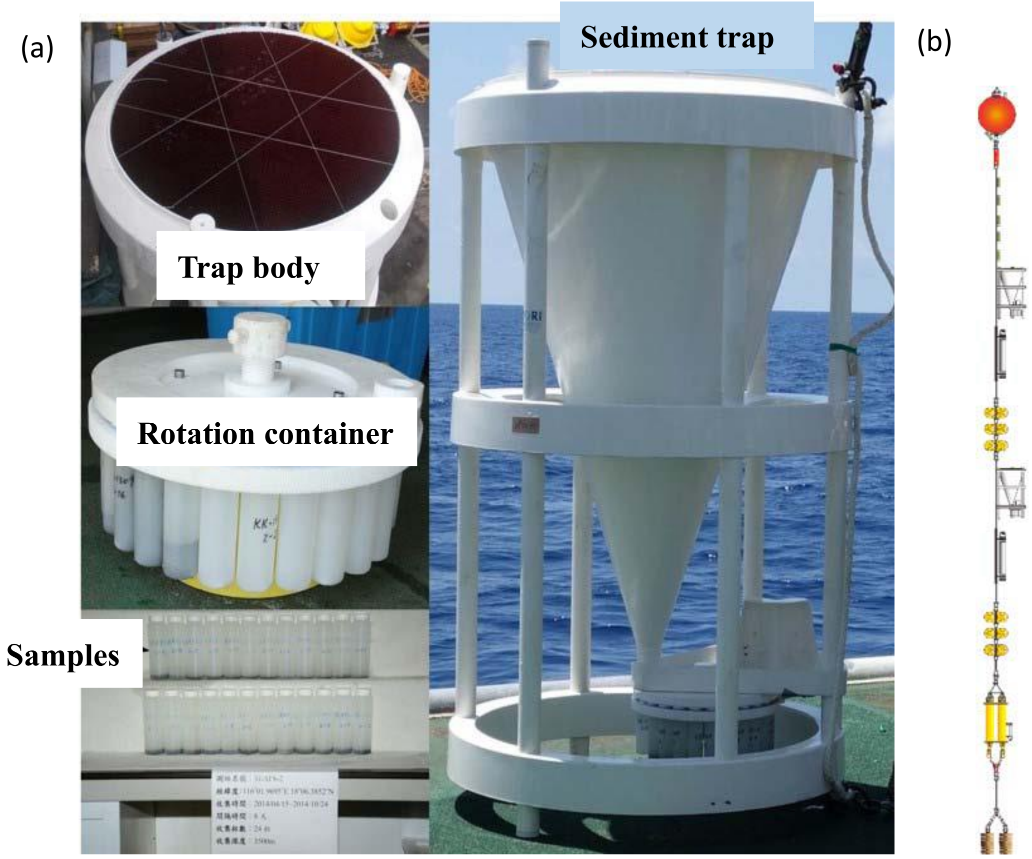

Figure S1. (a) The anchorage-type sediment trap (b) The two-series anchorage-type sediment trap

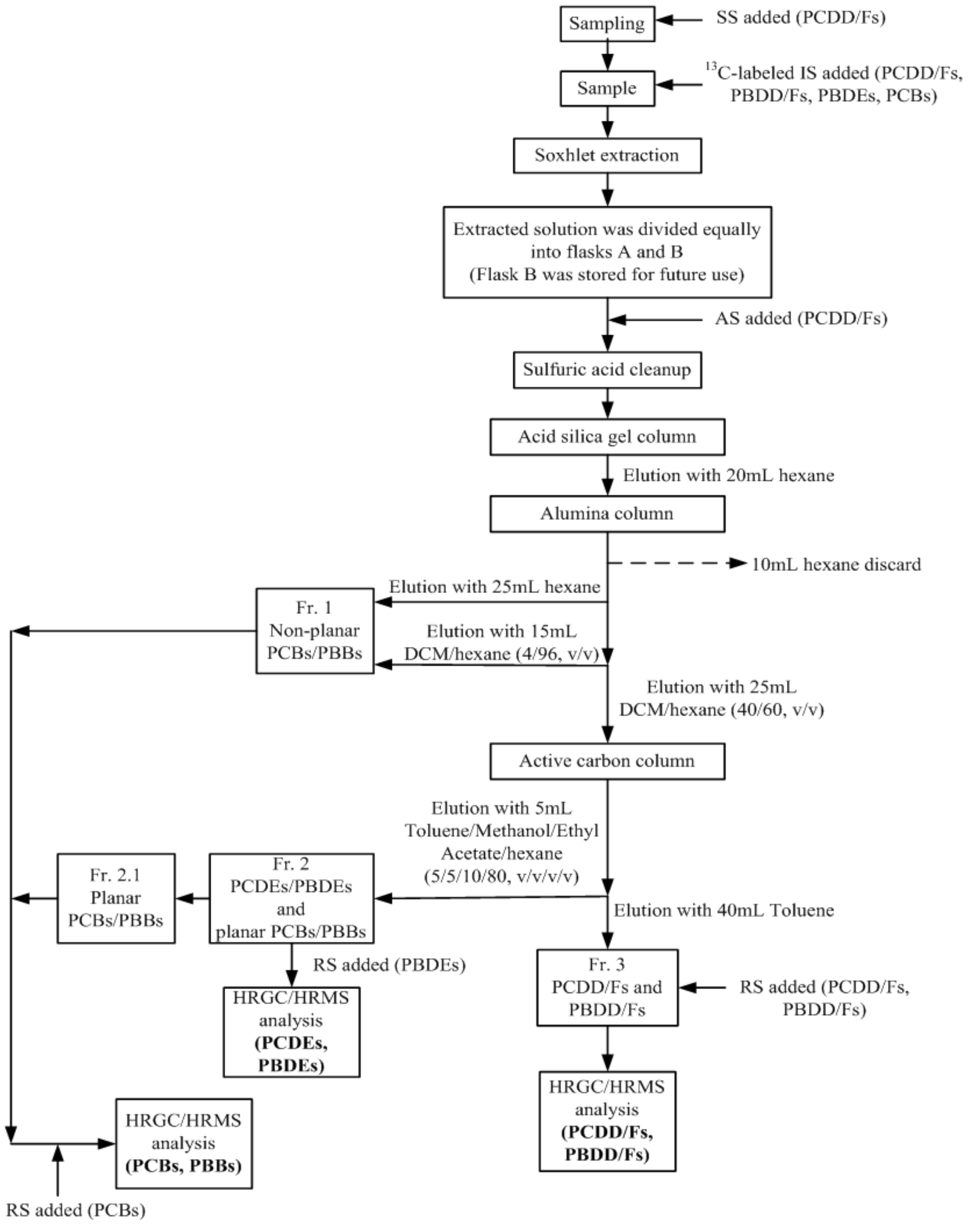

Figure S2 The analytical procedure of POPs in the present study [71]

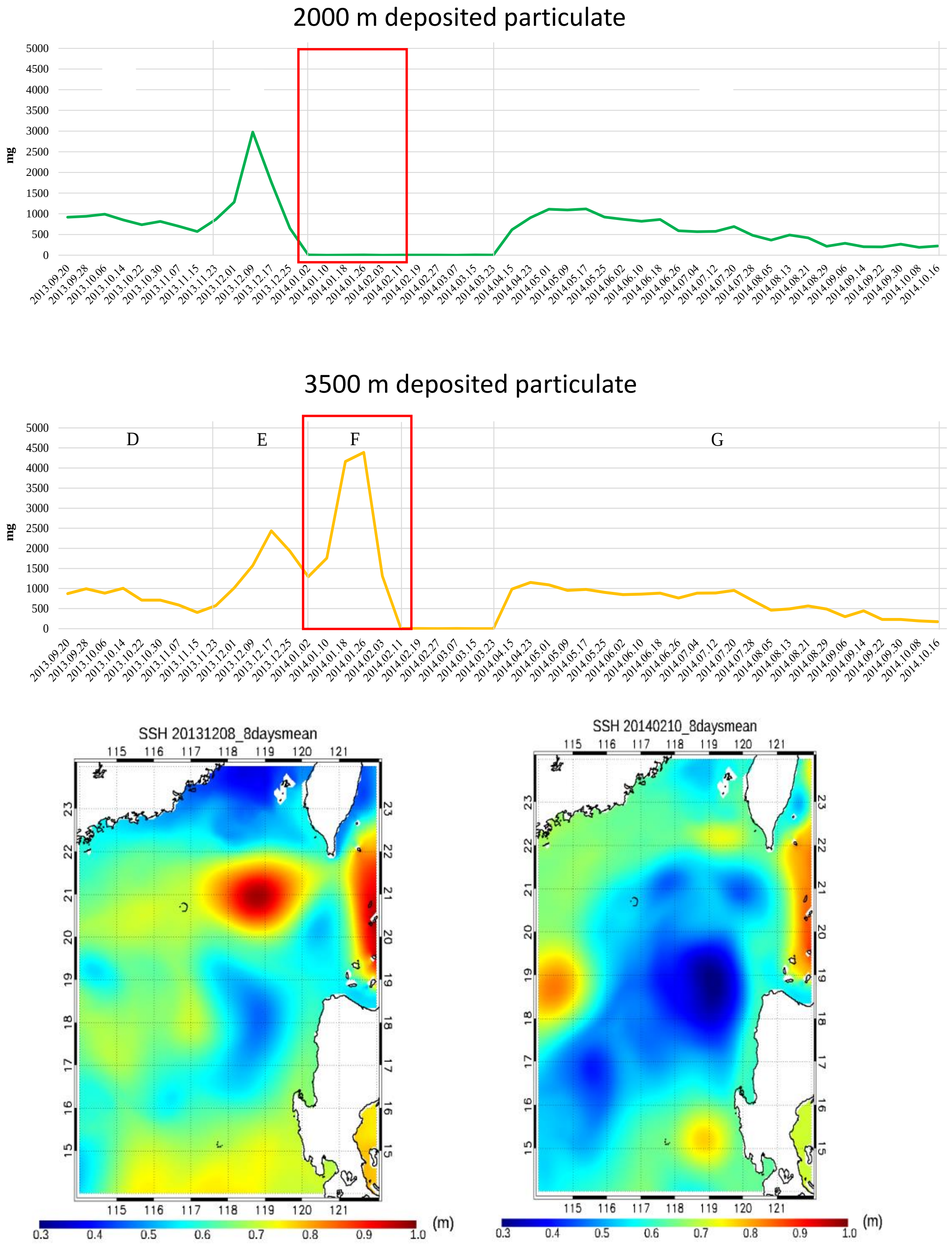

Figure S3. Sample C is collection of the deposited particulate between 2014/1/2 and 2014/2/11. Little mass is obtained at the depth of 2000 m during this duration possibly due to internal wave condition.

References

108. Zhang, K.; Wei, Y.-L.; Zeng, E.Y. A review of environmental and human exposure to persistent organic pollutants in the Pearl River Delta, South China. *Science of The Total Environment* **2013**, *463-464*, 1093-1110, doi:https://doi.org/10.1016/j.scitotenv.2012.10.104.
109. Zhang, Z.; Dai, M.; Hong, H.; Zhou, J.L.; Yu, G. Dissolved insecticides and polychlorinated biphenyls in the Pearl River Estuary and South China Sea. *Journal of Environmental Monitoring* **2002**, *4*, 922-928, doi:10.1039/B206891P.
110. Zhu, T.; Zhu, Y.; Liu, Y.; Deng, C.; Qi, X.; Wang, J.; Shen, Z.; Yin, D.; Liu, Y.; Sun, R.; et al. Polybrominated diphenyl ethers in water, suspended particulate matter, and sediment of reservoirs and their tributaries in Shenzhen, a mega city in South China. *Environ Sci Pollut Res Int* **2023**, *30*, 53524-53537, doi:10.1007/s11356-023-26066-6.
111. Xiong, J.; Li, G.; An, T.; Zhang, C.; Wei, C. Emission patterns and risk assessment of polybrominated diphenyl ethers and bromophenols in water and sediments from the Beijiang River, South China. *Environmental Pollution* **2016**, *219*, 596-603.
112. Guan, Y.-F.; Wang, J.-Z.; Ni, H.-G.; Zeng, E.Y. Riverine Inputs of Polybrominated Diphenyl Ethers from the Pearl River Delta (China) to the Coastal Ocean. *Environmental Science & Technology* **2007**, *41*, 6007-6013, doi:10.1021/es070782x.
113. Wurl, O.; Lam, P.K.S.; Obbard, J.P. Occurrence and distribution of polybrominated diphenyl ethers (PBDEs) in the dissolved and suspended phases of the sea-surface microlayer and seawater in Hong Kong, China. *Chemosphere* **2006**, *65*, 1660-1666, doi:https://doi.org/10.1016/j.chemosphere.2006.02.024.
114. Liu, Y.; Peng, P.a.; Li, X.; Zhang, S.; Ren, M. Polychlorinated dibenzo-p-dioxins and dibenzofurans (PCDD/Fs) in water and suspended particulate matter from the Xijiang River, China. *Journal of Hazardous Materials* **2008**, *152*, 40-47, doi:https://doi.org/10.1016/j.jhazmat.2007.06.071.
